# Supplementary material for: Near-perfect precise on-target editing of human hematopoietic stem and progenitor cells
Source: eLife. 2024 Jun 3;12:RP91288. doi: 10.7554/eLife.91288 (PMC11147503; doi:10.7554/eLife.91288)
Supplement: Supplementary file 1. — Figure and panel are indicated along with each pair-wise test. Where relevant the colony type or population for a given test is indicated. [file elife-91288-supp1.docx]

**Supplementary Table 1. Significance testing.** Figure and panel are indicated along with each pair-wise test. Where relevant the colony type or population for a given test is indicated.

| **Figure** | **Panel** | **Condition 1** | **Condition 2** | **Pvalue** | **FDR** | **Colony type / Population** |
| --- | --- | --- | --- | --- | --- | --- |
| 1 | c | SRSF2_AAV_yes_200 | SRSF2_AAV_yes_400 | 0.023277153 | 0.023277153 | NA |
| 1 | c | SRSF2_AAV_yes_400 | SRSF2_AAV_yes_800 | 0.012716956 | 0.023277153 | NA |
| 1 | d | SRSF2_ctrl_no_ 0 | SRSF2_AAV_no_200 | 0.21963085 | 0.241593935 | NA |
| 1 | d | SRSF2_ctrl_no_ 0 | SRSF2_AAV_yes_200 | 0.158741852 | 0.194017819 | NA |
| 1 | d | SRSF2_ctrl_no_ 0 | SRSF2_AAV_no_400 | 0.093385253 | 0.171206296 | NA |
| 1 | d | SRSF2_ctrl_no_ 0 | SRSF2_AAV_yes_400 | 0.068232666 | 0.150111866 | NA |
| 1 | d | SRSF2_ctrl_no_ 0 | SRSF2_AAV_no_800 | 0.002150054 | 0.023650592 | NA |
| 1 | d | SRSF2_ctrl_no_ 0 | SRSF2_AAV_yes_800 | 0.063494273 | 0.150111866 | NA |
| 1 | d | SRSF2_AAV_no_200 | SRSF2_AAV_yes_200 | 0.139663229 | 0.194017819 | NA |
| 1 | d | SRSF2_AAV_no_400 | SRSF2_AAV_yes_400 | 0.040511155 | 0.150111866 | NA |
| 1 | d | SRSF2_AAV_no_800 | SRSF2_AAV_yes_800 | 0.335216014 | 0.335216014 | NA |
| 1 | d | SRSF2_AAV_yes_200 | SRSF2_AAV_yes_400 | 0.152759319 | 0.194017819 | NA |
| 1 | d | SRSF2_AAV_yes_400 | SRSF2_AAV_yes_800 | 0.054599713 | 0.150111866 | NA |
| 1 | e | SRSF2_short_yes_1.00 | SRSF2_short_yes_2.50 | 0.022987461 | 0.045974921 | NA |
| 1 | e | SRSF2_short_yes_2.50 | SRSF2_short_yes_5.00 | 0.12410038 | 0.12410038 | NA |
| 1 | f | SRSF2_ctrl_no_0.00 | SRSF2_short_no_1.00 | 0.083705834 | 0.167411668 | NA |
| 1 | f | SRSF2_ctrl_no_0.00 | SRSF2_short_yes_1.00 | 0.14477643 | 0.183821244 | NA |
| 1 | f | SRSF2_ctrl_no_0.00 | SRSF2_short_no_2.50 | 0.116017299 | 0.17406597 | NA |
| 1 | f | SRSF2_ctrl_no_0.00 | SRSF2_short_yes_2.50 | 0.11604398 | 0.17406597 | NA |
| 1 | f | SRSF2_ctrl_no_0.00 | SRSF2_short_no_5.00 | 0.08124549 | 0.167411668 | NA |
| 1 | f | SRSF2_ctrl_no_0.00 | SRSF2_short_yes_5.00 | 0.063769856 | 0.167411668 | NA |
| 1 | f | SRSF2_ctrl_no_0.00 | SRSF2_long_no_0.25 | 0.057681145 | 0.167411668 | NA |
| 1 | f | SRSF2_ctrl_no_0.00 | SRSF2_long_yes_0.25 | 0.11508911 | 0.17406597 | NA |
| 1 | f | SRSF2_ctrl_no_0.00 | SRSF2_long_no_0.50 | 0.054620275 | 0.167411668 | NA |
| 1 | f | SRSF2_ctrl_no_0.00 | SRSF2_long_yes_0.50 | 0.070837822 | 0.167411668 | NA |
| 1 | f | SRSF2_ctrl_no_0.00 | SRSF2_long_no_1.00 | 0.036009341 | 0.167411668 | NA |
| 1 | f | SRSF2_ctrl_no_0.00 | SRSF2_long_yes_1.00 | 0.041960004 | 0.167411668 | NA |
| 1 | f | SRSF2_short_no_1.00 | SRSF2_short_yes_1.00 | 0.580156436 | 0.580156436 | NA |
| 1 | f | SRSF2_short_no_2.50 | SRSF2_short_yes_2.50 | 0.163396662 | 0.183821244 | NA |
| 1 | f | SRSF2_short_no_5.00 | SRSF2_short_yes_5.00 | 0.041119811 | 0.167411668 | NA |
| 1 | f | SRSF2_long_no_0.25 | SRSF2_long_yes_0.25 | 0.405690593 | 0.429554745 | NA |
| 1 | f | SRSF2_long_no_0.50 | SRSF2_long_yes_0.50 | 0.159350061 | 0.183821244 | NA |
| 1 | f | SRSF2_long_no_1.00 | SRSF2_long_yes_1.00 | 0.144754023 | 0.183821244 | NA |
| 2 | a | w/o mol | 0.5µM M3814 | 0.028352285 | 0.045363655 | NA |
| 2 | a | w/o mol | 0.5µM AZD7648 | 0.00649234 | 0.014454949 | NA |
| 2 | a | w/o mol | 5µM M3814 | 0.007227475 | 0.014454949 | NA |
| 2 | a | w/o mol | 5µM AZD7648 | 0.003966845 | 0.014454949 | NA |
| 2 | a | 0.5µM M3814 | 0.5µM AZD7648 | 0.001575337 | 0.012602695 | NA |
| 2 | a | 0.5µM AZD7648 | 5µM AZD7648 | 0.139940447 | 0.159931939 | NA |
| 2 | a | 0.5µM AZD7648 | 5µM M3814 | 0.235621749 | 0.235621749 | NA |
| 2 | a | 5µM AZD7648 | 5µM M3814 | 0.133223454 | 0.159931939 | NA |
| 2 | b | Donor only | w/o mol | 0.051358419 | 0.085597365 | NA |
| 2 | b | Donor only | 0.5µM M3814 | 0.035638736 | 0.071277472 | NA |
| 2 | b | Donor only | 0.5µM AZD7648 | 0.150783926 | 0.172700095 | NA |
| 2 | b | Donor only | 5µM M3814 | 0.002710079 | 0.013550395 | NA |
| 2 | b | Donor only | 5µM AZD7648 | 0.033072608 | 0.071277472 | NA |
| 2 | b | 0.5µM M3814 | 0.5µM AZD7648 | 0.00466545 | 0.0155515 | NA |
| 2 | b | 0.5µM M3814 | 5µM AZD7648 | 0.577112708 | 0.577112708 | NA |
| 2 | b | 0.5µM M3814 | 5µM M3814 | 0.155430085 | 0.172700095 | NA |
| 2 | b | 5µM M3814 | 5µM AZD7648 | 0.147738595 | 0.172700095 | NA |
| 2 | b | 0.5µM AZD7648 | 5µM AZD7648 | 0.001816811 | 0.013550395 | NA |
| 2 | c | p53 only_SRSF2_AAV_mutant | p53+RS-1_SRSF2_AAV_mutant | 0.482871408 | 0.482871408 | NA |
| 2 | c | p53 only_SRSF2_AAV_mutant | p53+AZD_SRSF2_AAV_mutant | 0.06039884 | 0.181196519 | NA |
| 2 | c | p53 only_SRSF2_AAV_mutant | p53+AZD+RS-1_SRSF2_AAV_mutant | 0.242830736 | 0.364246103 | NA |
| 2 | d | Donor only_SRSF2_AAV_ | p53 only_SRSF2_AAV_mutant | 0.740322668 | 0.740322668 | NA |
| 2 | d | Donor only_SRSF2_AAV_ | p53+RS-1_SRSF2_AAV_mutant | 0.156261873 | 0.25411052 | NA |
| 2 | d | Donor only_SRSF2_AAV_ | p53+AZD_SRSF2_AAV_mutant | 0.441147747 | 0.514672372 | NA |
| 2 | d | Donor only_SRSF2_AAV_ | p53+AZD+RS-1_SRSF2_AAV_mutant | 0.069699742 | 0.162632731 | NA |
| 2 | d | p53 only_SRSF2_AAV_mutant | p53+RS-1_SRSF2_AAV_mutant | 0.017514729 | 0.061301553 | NA |
| 2 | d | p53 only_SRSF2_AAV_mutant | p53+AZD_SRSF2_AAV_mutant | 0.181507515 | 0.25411052 | NA |
| 2 | d | p53+AZD_SRSF2_AAV_mutant | p53+AZD+RS-1_SRSF2_AAV_mutant | 0.010588436 | 0.061301553 | NA |
| 2 | g | p53+AZDlow_SRSF2_AAV_mutant | p53+AZDlow_SRSF2_short oligo_mutant | 1.48E-06 | 5.94E-06 | NA |
| 2 | g | p53+AZDlow_SRSF2_AAV_mutant | p53+AZDlow_SRSF2_short oligo_mutantNT | 0.02428539 | 0.03238052 | NA |
| 2 | g | p53+AZDlow_SRSF2_AAV_mutant | p53+AZDlow_SRSF2_short oligo_silent | 0.011248813 | 0.022497626 | NA |
| 2 | g | p53+AZDlow_SRSF2_short oligo_mutantNT | p53+AZDlow_SRSF2_short oligo_silent | 0.058467188 | 0.058467188 | NA |
| 2 | h | SRSF2OT1 Unedited | SRSF2OT1 Edited | 0.23025937 | 0.46051874 | NA |
| 2 | h | SRSF2OT1 Unedited | SRSF2OT1 Edited + AZD7648 | 0.054316581 | 0.325899489 | NA |
| 2 | h | SRSF2OT2 Unedited | SRSF2OT2 Edited | 0.189207494 | 0.46051874 | NA |
| 2 | h | SRSF2OT2 Unedited | SRSF2OT2 Edited + AZD7648 | 0.769245772 | 0.769245772 | NA |
| 2 | h | SRSF2OT3 Unedited | SRSF2OT3 Edited | 0.459381957 | 0.551258348 | NA |
| 2 | h | SRSF2OT3 Unedited | SRSF2OT3 Edited + AZD7648 | 0.449937162 | 0.551258348 | NA |
| 3 | a | LT-HSC |  | 0.770584266 | 0.770584266 | NA |
| 3 | a | IT-HSCs |  | 0.234177964 | 0.664959451 | NA |
| 3 | a | MPP |  | 0.477767032 | 0.664959451 | NA |
| 3 | a | Prog |  | 0.498719588 | 0.664959451 | NA |
| 3 | b | Ctrl elec | donor only | 0.348617206 | 0.398419665 | LT-HSC |
| 3 | b | Ctrl elec | donor only | 0.107055326 | 0.171288522 | IT-HSC |
| 3 | b | Ctrl elec | donor only | 0.176332651 | 0.235110201 | MPP |
| 3 | b | Ctrl elec | donor only | 0.088854072 | 0.171288522 | Prog |
| 3 | b | Ctrl elec | Silent | 0.084251875 | 0.171288522 | LT-HSC |
| 3 | b | Ctrl elec | Silent | 0.47964894 | 0.47964894 | IT-HSC |
| 3 | b | Ctrl elec | Silent | 0.014739174 | 0.058956698 | MPP |
| 3 | b | Ctrl elec | Silent | 0.000888205 | 0.007105639 | Prog |
| 3 | d | Ctrl elec | sil w/o RNP | 0.062919995 | 0.067769255 | NA |
| 3 | d | Ctrl elec | sil w RNP | 0.012072095 | 0.036216285 | NA |
| 3 | d | sil w/o RNP | sil w RNP | 0.067769255 | 0.067769255 | NA |
| 3 | e | Ctrl elec | sil w/o RNP | 0.118180694 | 0.531813123 | GEMM |
| 3 | e | Ctrl elec | sil w RNP | 0.588118599 | 0.731982001 | GEMM |
| 3 | e | sil w/o RNP | sil w RNP | 0.013453235 | 0.121079113 | GEMM |
| 3 | e | Ctrl elec | sil w/o RNP | 0.251099079 | 0.731982001 | GM |
| 3 | e | Ctrl elec | sil w RNP | 0.530924473 | 0.731982001 | GM |
| 3 | e | sil w/o RNP | sil w RNP | 0.608350943 | 0.731982001 | GM |
| 3 | e | Ctrl elec | sil w/o RNP | 0.659028761 | 0.731982001 | E |
| 3 | e | Ctrl elec | sil w RNP | 0.465740918 | 0.731982001 | E |
| 3 | e | sil w/o RNP | sil w RNP | 0.731982001 | 0.731982001 | E |
| 1-S1 | a | 30.5_- | 30.5_+ | 0.005011906 | 0.020047625 | NA |
| 1-S1 | a | 30.5_- | 61.0_- | 0.046130418 | 0.084414376 | NA |
| 1-S1 | a | 30.5_- | 61.0_+ | 0.063310782 | 0.084414376 | NA |
| 1-S1 | a | 61.0_- | 61.0_+ | 0.482327699 | 0.482327699 | NA |
| 1-S1 | b | 30.5_- | 30.5_+ | 0.009085768 | 0.012114358 | NA |
| 1-S1 | b | 30.5_- | 61.0_- | 0.390896739 | 0.390896739 | NA |
| 1-S1 | b | 30.5_- | 61.0_+ | 0.000985007 | 0.00328815 | NA |
| 1-S1 | b | 61.0_- | 61.0_+ | 0.001644075 | 0.00328815 | NA |
| 1-S1 | a | 30.5_- | 30.5_+ | 0.113475776 | 0.151301035 | NA |
| 1-S1 | a | 30.5_- | 61.0_- | 0.468501111 | 0.468501111 | NA |
| 1-S1 | a | 30.5_- | 61.0_+ | 0.04124467 | 0.146545815 | NA |
| 1-S1 | a | 61.0_- | 61.0_+ | 0.073272907 | 0.146545815 | NA |
| 1-S1 | d | 30.5_- | 30.5_+ | 0.001208839 | 0.002417678 | NA |
| 1-S1 | d | 30.5_- | 61.0_- | 0.472774519 | 0.472774519 | NA |
| 1-S1 | d | 30.5_- | 61.0_+ | 3.87E-05 | 0.000154923 | NA |
| 1-S1 | d | 61.0_- | 61.0_+ | 0.003437052 | 0.004582736 | NA |
| 2-S1 | a | p53 only_SF3B1_AAV_mutant | p53+RS-1_SF3B1_AAV_mutant | 0.893320553 | 0.893320553 | NA |
| 2-S1 | a | p53 only_SF3B1_AAV_mutant | p53+AZD_SF3B1_AAV_mutant | 2.13E-06 | 8.53E-06 | NA |
| 2-S1 | a | p53 only_SF3B1_AAV_mutant | p53+AZD+RS-1_SF3B1_AAV_mutant | 0.002997226 | 0.005994452 | NA |
| 2-S1 | a | p53+AZD_SF3B1_AAV_mutant | p53+AZD+RS-1_SF3B1_AAV_mutant | 0.007684271 | 0.010245695 | NA |
| 2-S1 | b | p53 only_SF3B1_AAV_mutant | p53+RS-1_SF3B1_AAV_mutant | 0.058629221 | 0.100709321 | NA |
| 2-S1 | b | p53 only_SF3B1_AAV_mutant | p53+AZD_SF3B1_AAV_mutant | 0.76158459 | 0.76158459 | NA |
| 2-S1 | b | p53 only_SF3B1_AAV_mutant | p53+AZD+RS-1_SF3B1_AAV_mutant | 0.049168908 | 0.100709321 | NA |
| 2-S1 | b | p53+AZD_SF3B1_AAV_mutant | p53+AZD+RS-1_SF3B1_AAV_mutant | 0.075531991 | 0.100709321 | NA |
