# Supplementary material for: Near-perfect precise on-target editing of human hematopoietic stem and progenitor cells
Source: eLife. 2024 Jun 3;12:RP91288. doi: 10.7554/eLife.91288 (PMC11147503; doi:10.7554/eLife.91288)
Supplement: Supplementary file 2. [file elife-91288-supp2.docx]

**Annotated DNA sequences**

**Silent mutation**

**Mutation**

Synthetic Intron/Inserted Sequence

Guide RNA complementary sequence

SRSF2 Silent ssODN:

t*g*gacggccgcgagctgcgggtgcaaatggcgcgctacggccgccc**T**cc**A**ga**T**tcacaccacagccgccggggaccgccaccccgcag*g*t

SRSF2 P95H ssODN:

t*g*gacggccgcgagctgcgggtgcaaatggcgcgctacggccgcc**AT**ccggactcacaccacagccgccggggaccgccaccccgcag*g*t

SRSF2 long ssODN donor

ttcacgacaagcgcgacgctgaggacgctatggatgccatggacggggccgtgctggacggccgcgagctgcgggtgcaaatggcgcgctacggccgcc**AT**ccggactcacaccacagccgccggggaccgccaccccgcaggtacgggggcggtggctacggacgccggagccgcaggtaaacggggctgaggggaccg

SRSF2 P95H ssODN with additional silent mutations:

t*g*gacggccgcgagctgcgggtgcaaatggcgcgctacggccgcc**AT**cc**A**ga**T**tcacaccacagccgccggggaccgccaccccgcag*g*t

SRSF2 P95H AAV donor sequence:

cctgcaggcagctgcgcgctcgctcgctcactgaggccgcccgggcgtcgggcgacctttggtcgcccggcctcagtgagcgagcgagcgcgcagagagggagtggccaactccatcactaggggttcctgcggcctctagACCGGCGTCCGTGCTGTTCTGCGGCaaggcctttcccagtgtccccacgcggaaggcaactgcctgagaggcgcggcgtcgcaccgcccagagctgaggaagccggcgccagttcgcggggctccgggccgccactcagagctatgagctacggccgcccccctcccgatgtggagggtatgacctccctcaaggtggacaacctgacctaccgcacctcgcccgacacgctgaggcgcgtcttcgagaagtacgggcgcgtcggcgacgtgtacatcccgcgggaccgctacaccaaggagtcccgcggcttcgccttcgttcgctttcacgacaagcgcgacgctgaggacgctatggatgccatggacggggccgtgctggacggccgcgagctgcgggtgcaaatggcgcgctacggccgcc**A**cccggtAAGTgAAAAAagcatagctctaaaacTGCTTCGCTACTGCATCGGCCGGGAATCGAACCCGGGCCGCCCGCGTGGCAGGCGAGCATTCTACCACTGAACCACCGATGCTACTAACTCGAGagTTCTTTCTTTCTTTCACAGgactcacaccacagccgccggggaccgccaccccgcaggtacgggggcggtggctacggacgccggagccgcaggtaaacggggctgaggggaccgcgggaggcggggcggggcgcgcgggaggcccgggcgacctcacaaaggtccgcggcgaagcacgtggtgcgggcccggacggggcgggggtgcacgccgcgtctcgcgaccctccggccaccccgcgagcttccgccgtctgcgacccgggagtggccggggtgtgggcggcgcggggcggaggaccccgcctcgcgactggggaaatggcgtctggcggcgagataatggcggcctgggcgggagcgcgcgggcggggccggccccgctgcctggaattaaccccgctgtgcttgctcgtcccgcccgcagccctaggcggcgtcgccgcagccgatcccggagtcggagtcgttccaggtctcgcagccgatctcgctacagCCGCTCGAAGTCTCGGTCCCGCTAGTgcggccgcaggaacccctagtgatggagttggccactccctctctgcgcgctcgctcgctcactgaggccgggcgaccaaaggtcgcccgacgcccgggctttgcccgggcggcctcagtgagcgagcgagcgcgca

SF3B1 K700E AAV donor sequence

GAGCTTTTGCTGTTGTAGCCTCtgccctgggcattccttctttattgcccttcttaaaagctgtgtgcaaaagcaagaagtcctggcaagcgagacacactggtattaagattgtacaacagatagctattcttatgggctgtgccatcttgccacatcttagaagtttagttgaaatcattgaacatggtaagttgtaatgtaactttgtctttttttttttttctttaggagacagggtcttactatgttgcccagactggactcaaacctttgggctcaagtgatcctcctgctcagcctccttagtagttgggactagaggtacacacacagcctgtccatgtttaataggacagctgtcctaaaattTGGGCTACTGATTTGGGGAGataaatggaaaggcatagctctacaaactatagattttatgatgggtttgttatattatctgctgacaggctatggttcatgttttgcttttacctaattttgtttaatgtgaacatattctgcagtttggctgaatagttgatatattgagagaatctggatgatattgtgtaacttaggtaatgttggAAAAAagcatagctctaaaacTGCTTCGCTACTGCATCGGCCGGGAATCGAACCCGGGCCGCCCGCGTGGCAGGCGAGCATTCTACCACTGAACCACCGATGCggcatagttaaaacctgtgtttg**C**ttttgtaggtcttgt**T**gatgagca**A**cag**G**aagttcggaccatcagtgctttggccattgctgccttggctgaagcagcaactccttatggtatcgaatcttttgattctgtgttaaagcctttatggAAGGGTATCCGCCAACACAGaggaaaggtaaatccaccaattaccttttgatttatcttcattaaagttaaggcgacataaatctaaattactaaagtacatatattttttatttaaaaatagggtttggctgctttcttgaaggctattgggtatcttattcctcttatggatgcagaatatgccaactactatactagagaagtgatgttaatccttattcgagaattccagtctcctgatgaggaaatgaaaaaaattgtgctgaaggtaattattccagatttgttaatgtaaaCTGGATATGTTTCATGGTTCTAACATAGT
