# Supplementary figures and images for: Near-perfect precise on-target editing of human hematopoietic stem and progenitor cells
Source: eLife. 2024 Jun 3;12:RP91288. doi: 10.7554/eLife.91288 (PMC11147503; doi:10.7554/eLife.91288)

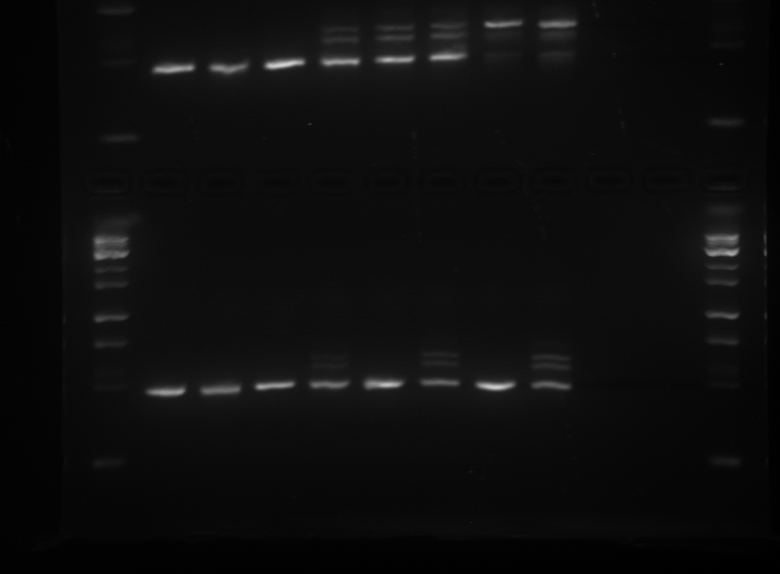

Supplement: Source data 1. — Data file for the raw gels compiled in Source data 2. [file elife-91288-data1.zip › Source Data – File 1 - Raw Gel Images/Figure 1C AAV MOI replicate 1.tif]

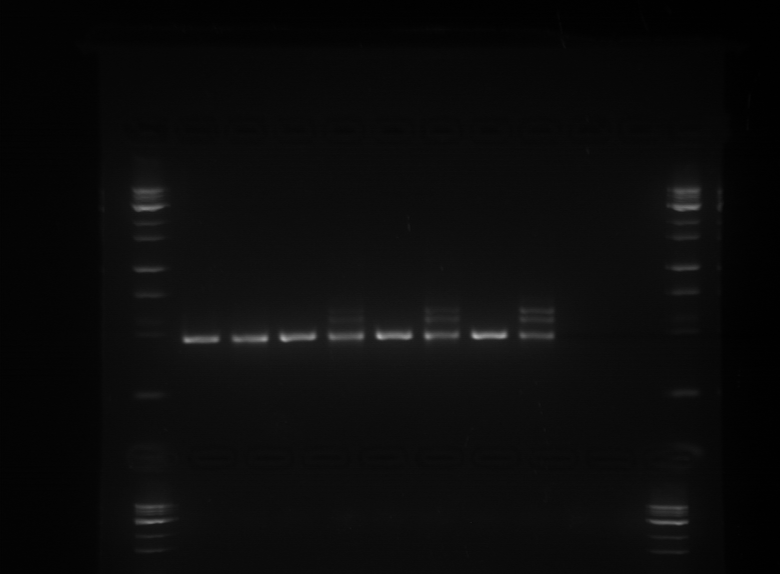

Supplement: Source data 1. — Data file for the raw gels compiled in Source data 2. [file elife-91288-data1.zip › Source Data – File 1 - Raw Gel Images/Figure 1C AAV MOI replicate 2.tif]

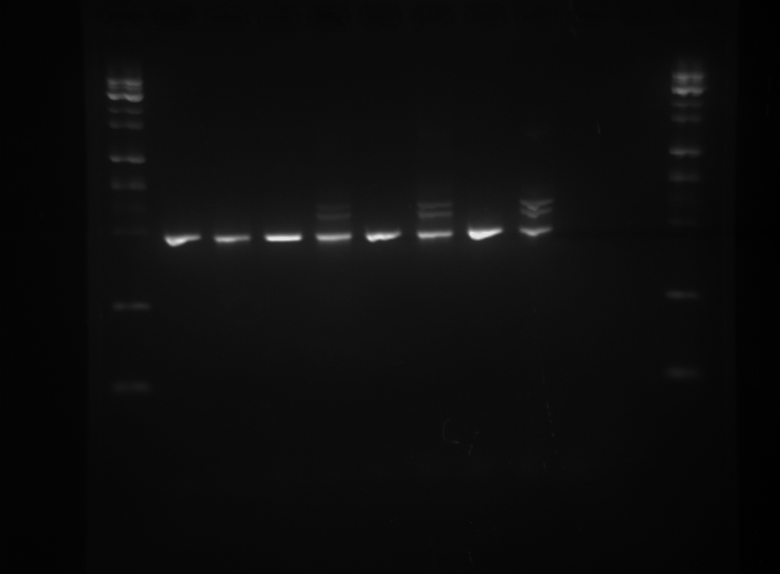

Supplement: Source data 1. — Data file for the raw gels compiled in Source data 2. [file elife-91288-data1.zip › Source Data – File 1 - Raw Gel Images/Figure 1C AAV MOI replicate 3.tif]

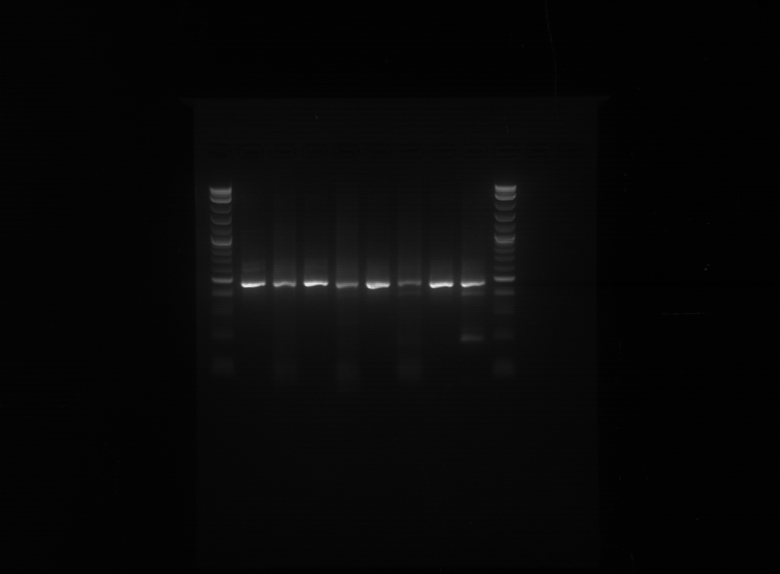

Supplement: Source data 1. — Data file for the raw gels compiled in Source data 2. [file elife-91288-data1.zip › Source Data – File 1 - Raw Gel Images/Figure 1E Replicate 1 for short donor.tif]

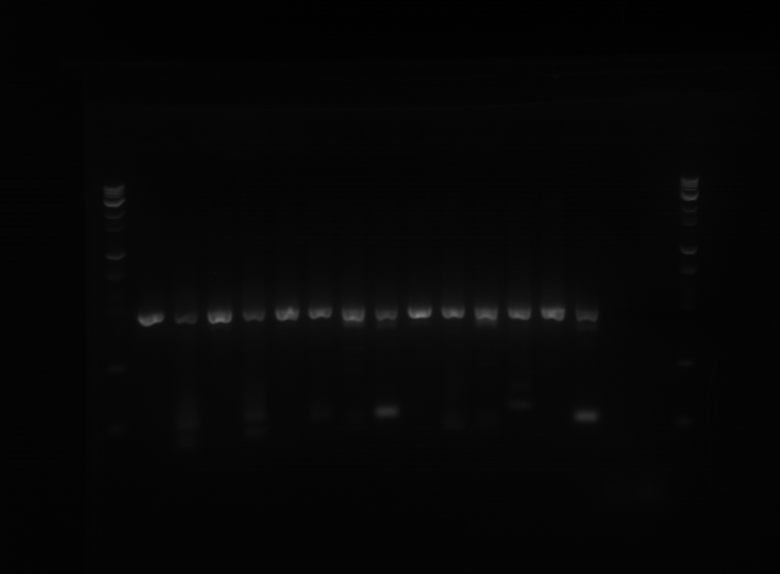

Supplement: Source data 1. — Data file for the raw gels compiled in Source data 2. [file elife-91288-data1.zip › Source Data – File 1 - Raw Gel Images/Figure 1E Replicate 2 for short donor and 1 for long donor.tif]

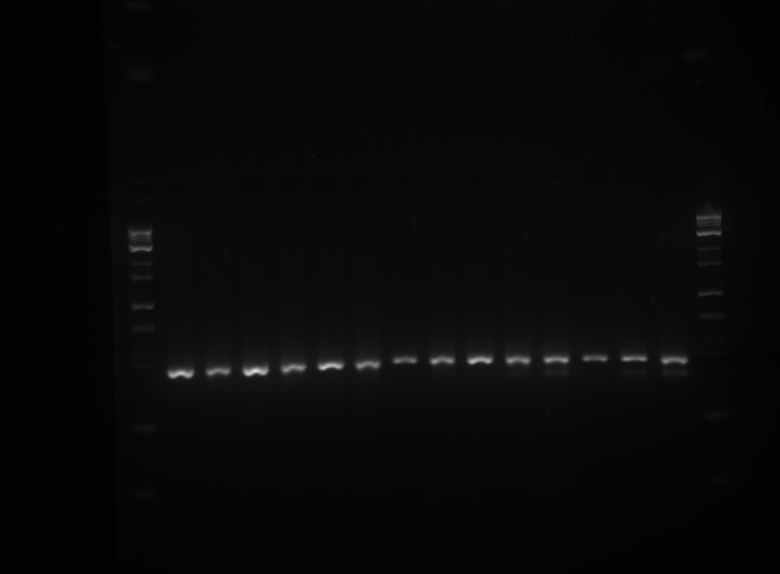

Supplement: Source data 1. — Data file for the raw gels compiled in Source data 2. [file elife-91288-data1.zip › Source Data – File 1 - Raw Gel Images/Figure 1E Replicate 3 for long donor.tif]

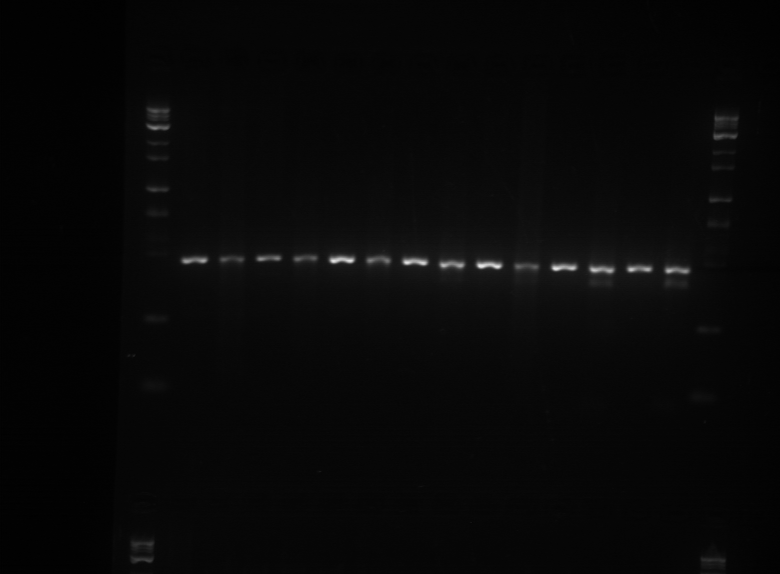

Supplement: Source data 1. — Data file for the raw gels compiled in Source data 2. [file elife-91288-data1.zip › Source Data – File 1 - Raw Gel Images/Figure 1E Replicate 3 for short donor and 2 for long donor.tif]

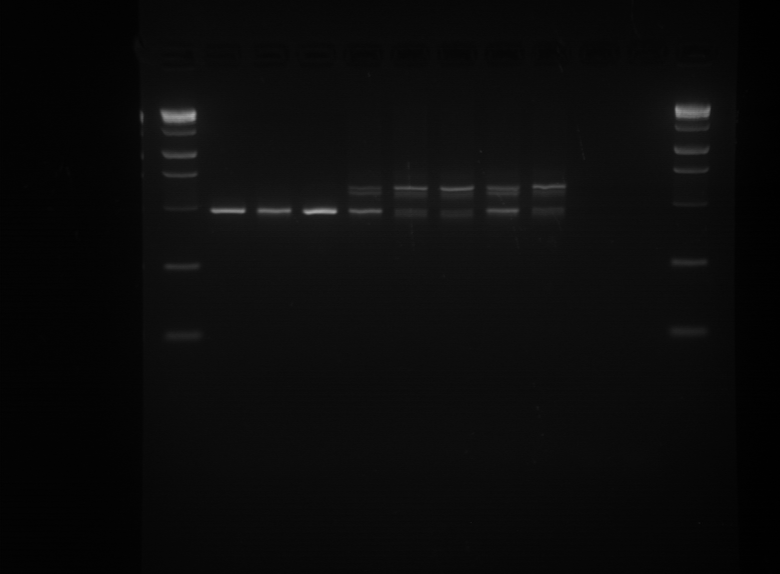

Supplement: Source data 1. — Data file for the raw gels compiled in Source data 2. [file elife-91288-data1.zip › Source Data – File 1 - Raw Gel Images/Figure 2A AZD7648 vs M3814 replicate 1.tif]

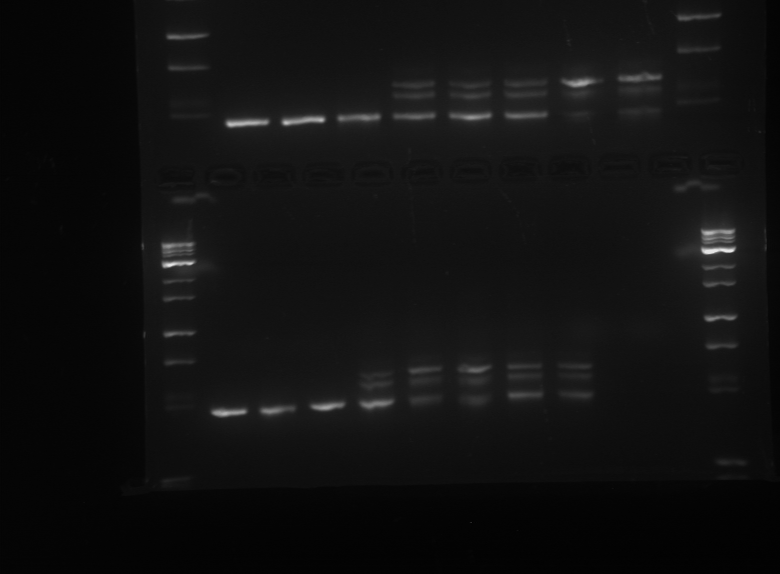

Supplement: Source data 1. — Data file for the raw gels compiled in Source data 2. [file elife-91288-data1.zip › Source Data – File 1 - Raw Gel Images/Figure 2A AZD7648 vs M3814 replicate 2.tif]

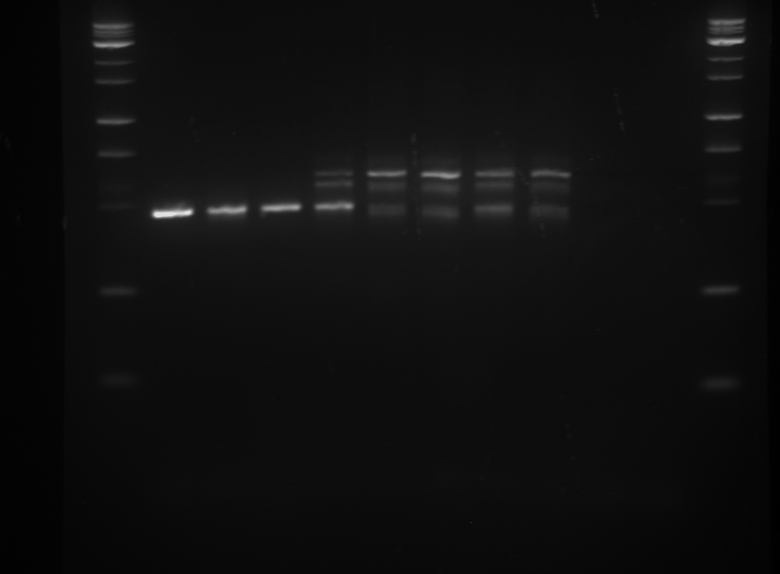

Supplement: Source data 1. — Data file for the raw gels compiled in Source data 2. [file elife-91288-data1.zip › Source Data – File 1 - Raw Gel Images/Figure 2A AZD7648 vs M3814 replicate 3.tif]

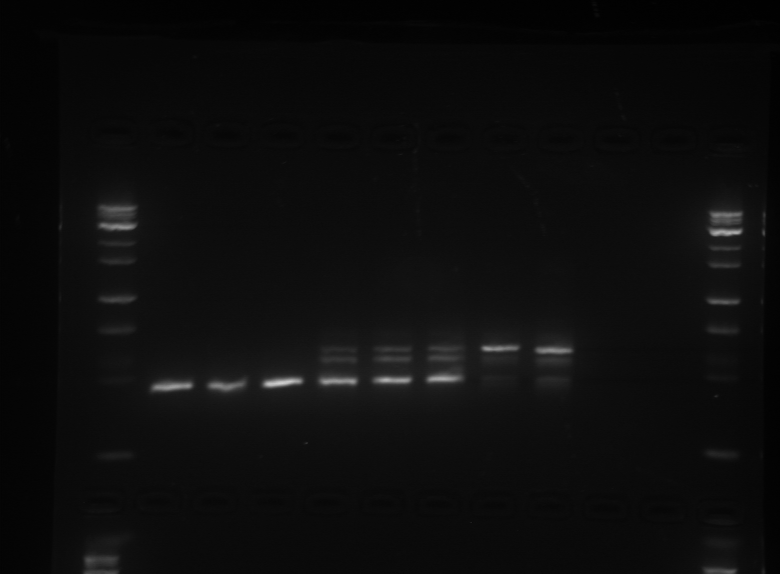

Supplement: Source data 1. — Data file for the raw gels compiled in Source data 2. [file elife-91288-data1.zip › Source Data – File 1 - Raw Gel Images/Figure 2C AZD7648 and RS1 additive effect assay replicate 1.tif]

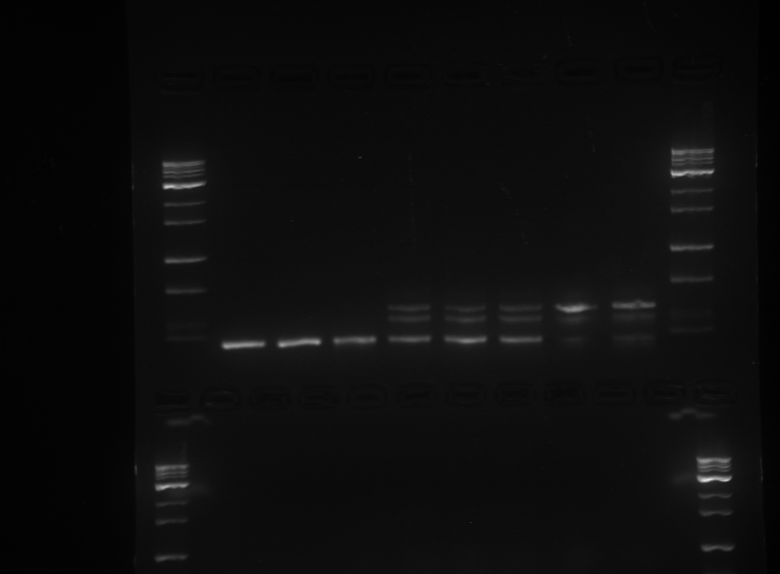

Supplement: Source data 1. — Data file for the raw gels compiled in Source data 2. [file elife-91288-data1.zip › Source Data – File 1 - Raw Gel Images/Figure 2C AZD7648 and RS1 additive effect assay replicate 2.tif]

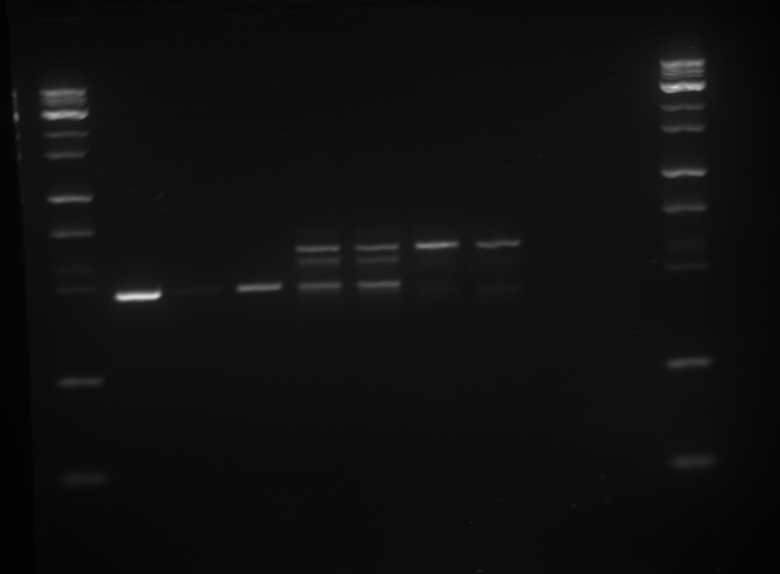

Supplement: Source data 1. — Data file for the raw gels compiled in Source data 2. [file elife-91288-data1.zip › Source Data – File 1 - Raw Gel Images/Figure 2C AZD7648 and RS1 additive effect assay replicate 3.tif]

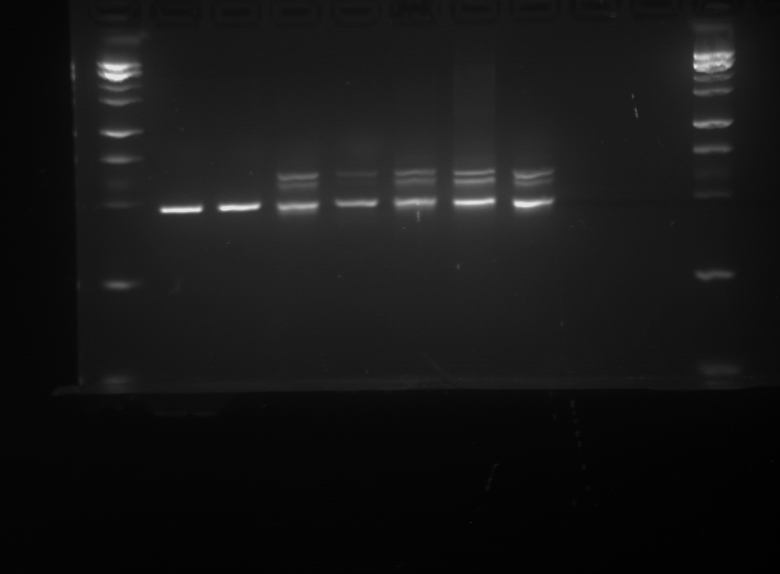

Supplement: Source data 1. — Data file for the raw gels compiled in Source data 2. [file elife-91288-data1.zip › Source Data – File 1 - Raw Gel Images/Figure 3A Integration assessment in all 4 sub-populations replicate 1.tif]

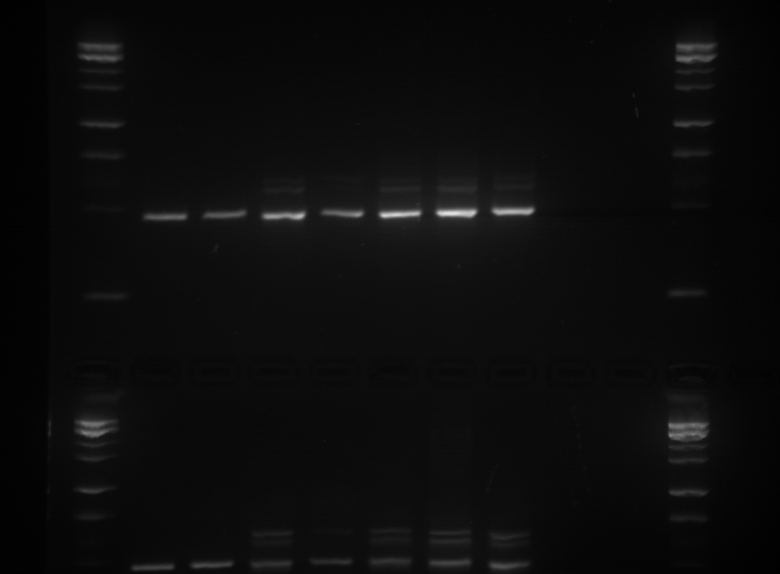

Supplement: Source data 1. — Data file for the raw gels compiled in Source data 2. [file elife-91288-data1.zip › Source Data – File 1 - Raw Gel Images/Figure 3A Integration assessment in all 4 sub-populations replicate 2.tif]

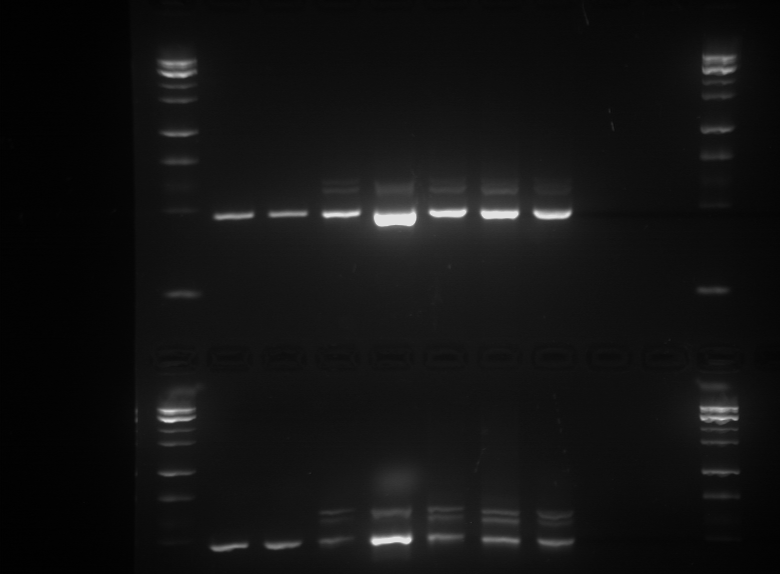

Supplement: Source data 1. — Data file for the raw gels compiled in Source data 2. [file elife-91288-data1.zip › Source Data – File 1 - Raw Gel Images/Figure 3A Integration assessment in all 4 sub-populations replicate 3.tif]

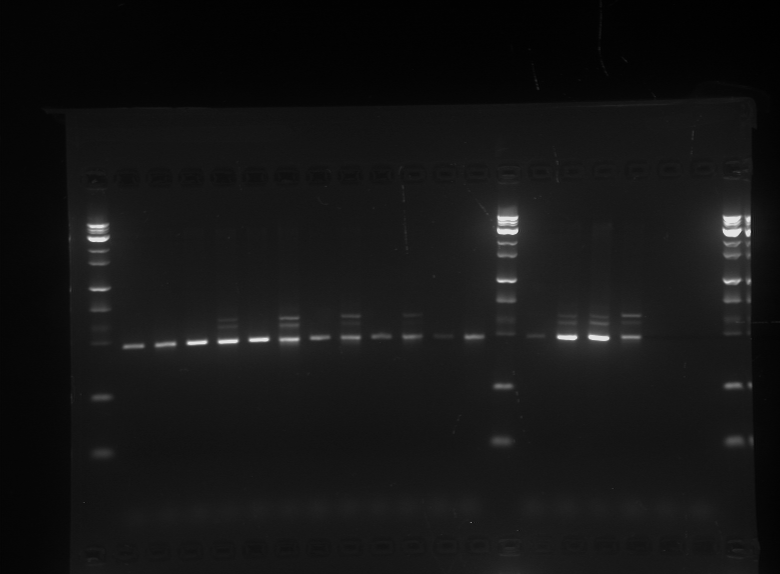

Supplement: Source data 1. — Data file for the raw gels compiled in Source data 2. [file elife-91288-data1.zip › Source Data – File 1 - Raw Gel Images/Figure 4B Integration rate in the bulk assessment replicate 1.tif]

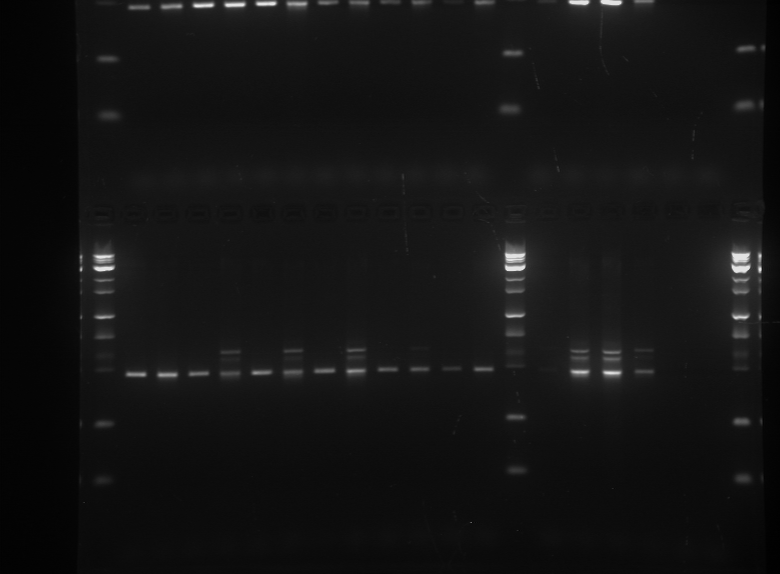

Supplement: Source data 1. — Data file for the raw gels compiled in Source data 2. [file elife-91288-data1.zip › Source Data – File 1 - Raw Gel Images/Figure 4B Integration rate in the bulk assessment replicate 2.tif]

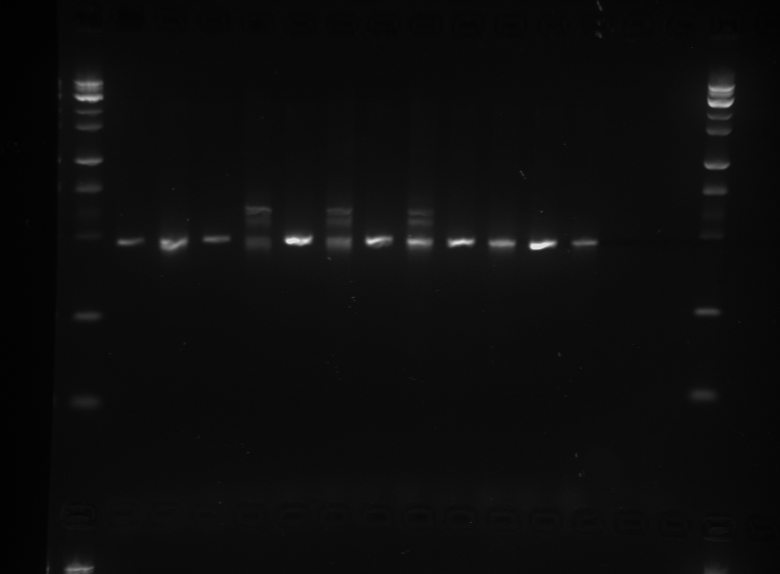

Supplement: Source data 1. — Data file for the raw gels compiled in Source data 2. [file elife-91288-data1.zip › Source Data – File 1 - Raw Gel Images/Figure 4B Integration rate in the bulk assessment replicate 3.tif]

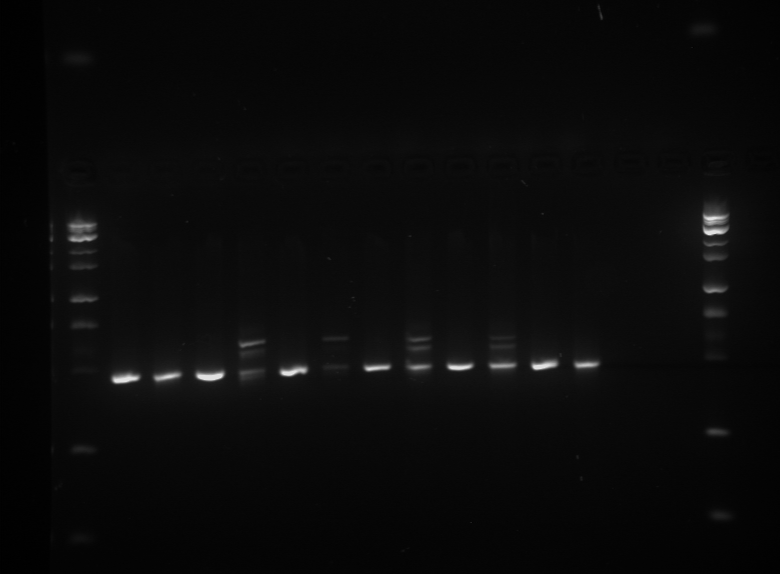

Supplement: Source data 1. — Data file for the raw gels compiled in Source data 2. [file elife-91288-data1.zip › Source Data – File 1 - Raw Gel Images/Figure 4B Integration rate in the bulk assessment replicate 4.tif]

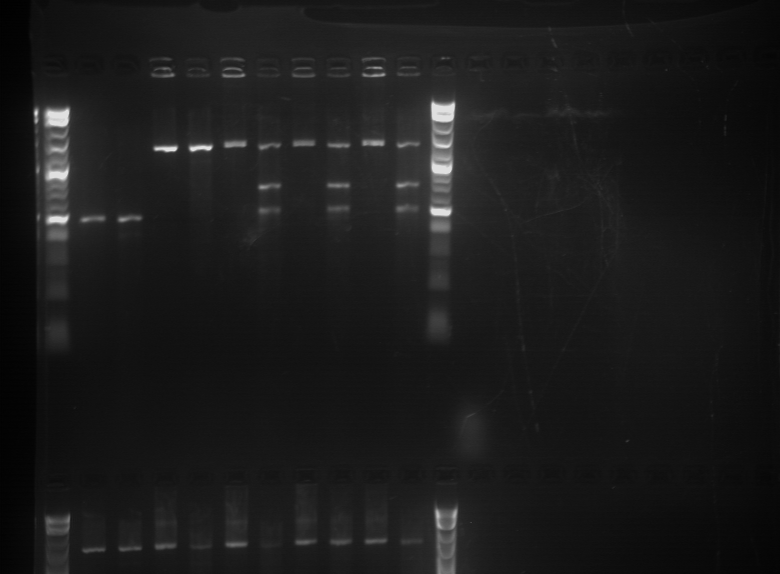

Supplement: Source data 1. — Data file for the raw gels compiled in Source data 2. [file elife-91288-data1.zip › Source Data – File 1 - Raw Gel Images/Figure S1A SRSF2 replicate 1.tif]

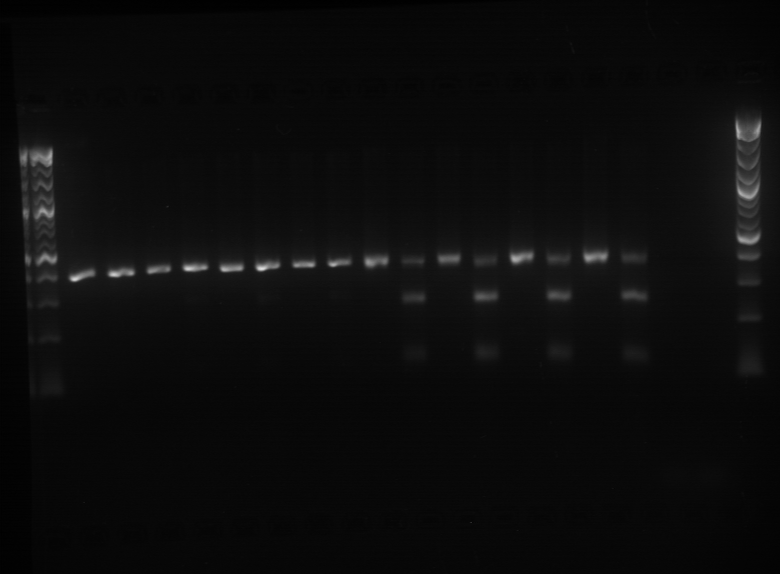

Supplement: Source data 1. — Data file for the raw gels compiled in Source data 2. [file elife-91288-data1.zip › Source Data – File 1 - Raw Gel Images/Figure S1A SRSF2 replicate 2.tif]

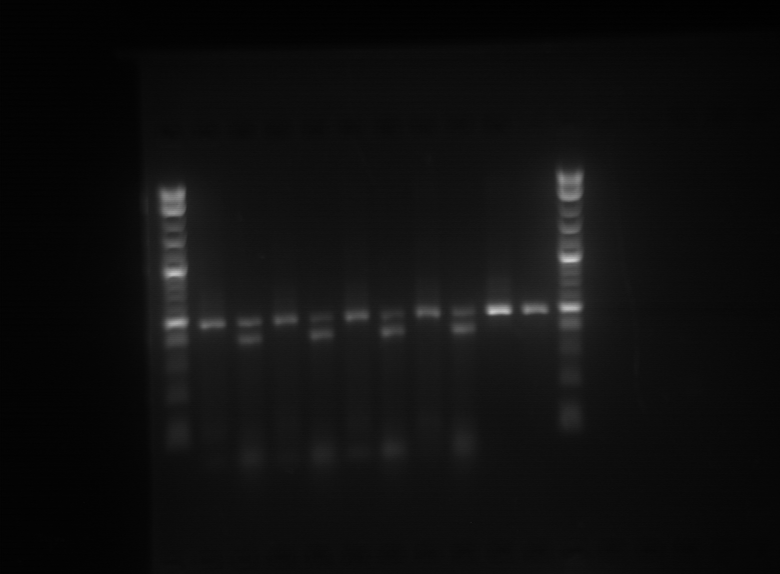

Supplement: Source data 1. — Data file for the raw gels compiled in Source data 2. [file elife-91288-data1.zip › Source Data – File 1 - Raw Gel Images/Figure S1A SRSF2 replicate 3.tif]

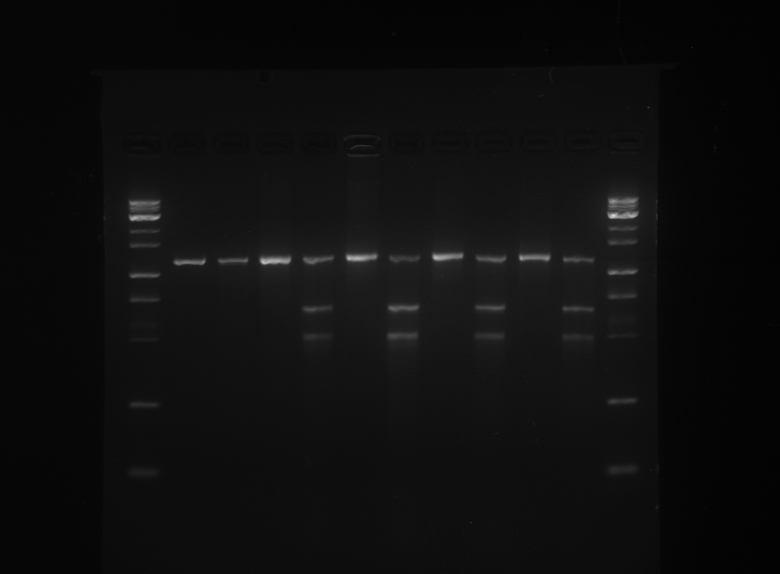

Supplement: Source data 1. — Data file for the raw gels compiled in Source data 2. [file elife-91288-data1.zip › Source Data – File 1 - Raw Gel Images/Figure S1A SRSF2 replicate 4.tif]

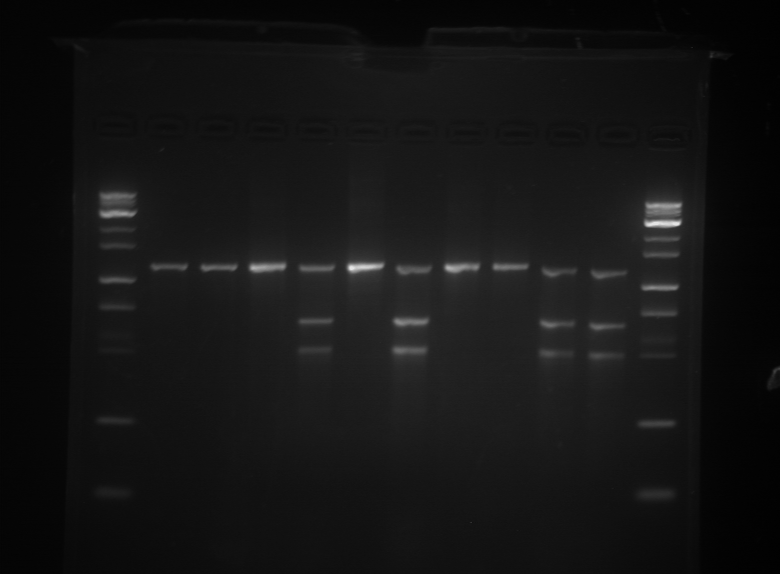

Supplement: Source data 1. — Data file for the raw gels compiled in Source data 2. [file elife-91288-data1.zip › Source Data – File 1 - Raw Gel Images/Figure S1A SRSF2 replicate 5.tif]

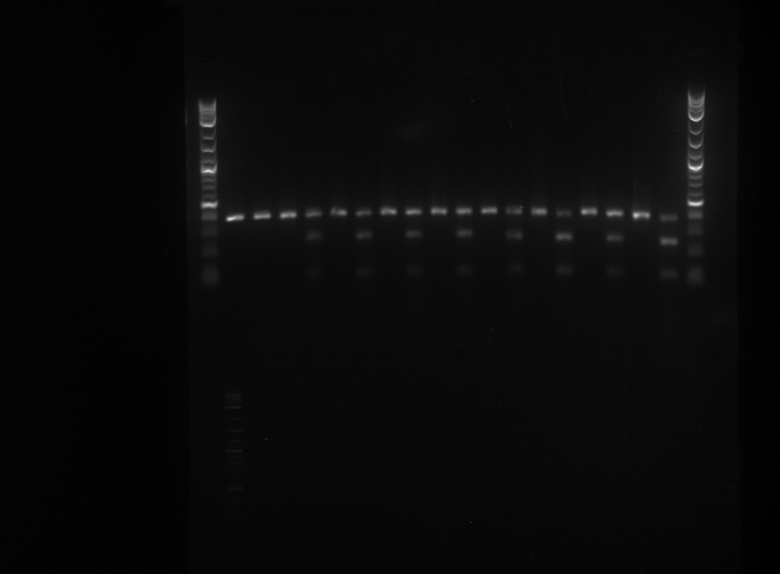

Supplement: Source data 1. — Data file for the raw gels compiled in Source data 2. [file elife-91288-data1.zip › Source Data – File 1 - Raw Gel Images/Figure S1C RNP cutting efficiency for SF3B1 gene replicate 1 and 2.tif]

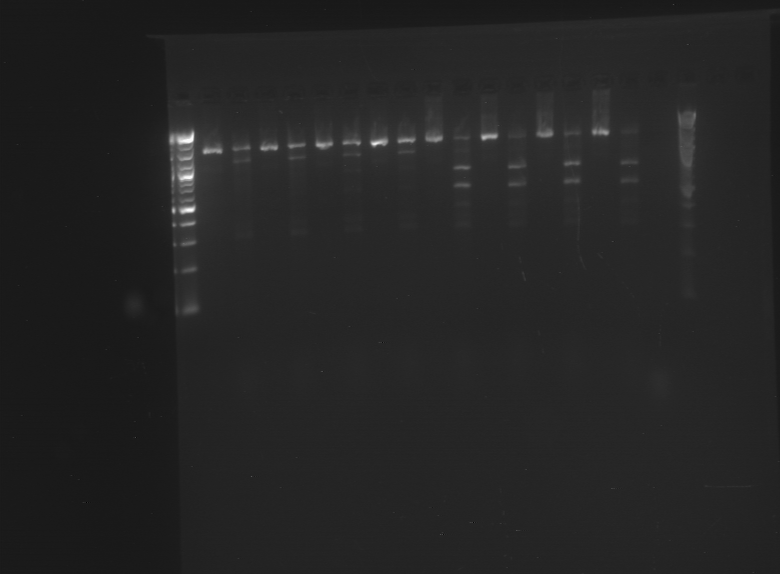

Supplement: Source data 1. — Data file for the raw gels compiled in Source data 2. [file elife-91288-data1.zip › Source Data – File 1 - Raw Gel Images/Figure S1C RNP cutting efficiency for SF3B1 gene replicate 3.tif]

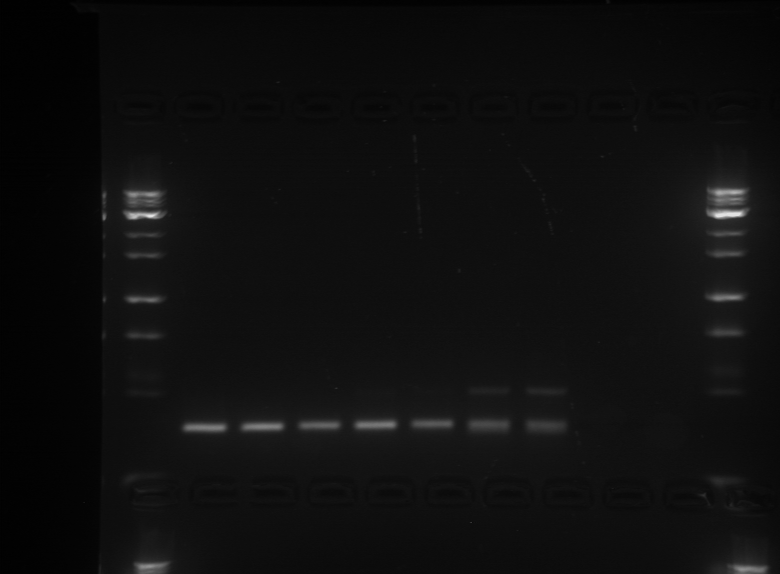

Supplement: Source data 1. — Data file for the raw gels compiled in Source data 2. [file elife-91288-data1.zip › Source Data – File 1 - Raw Gel Images/Figure S3A Integration rate for SF3B1 gene replicate 1.tif]

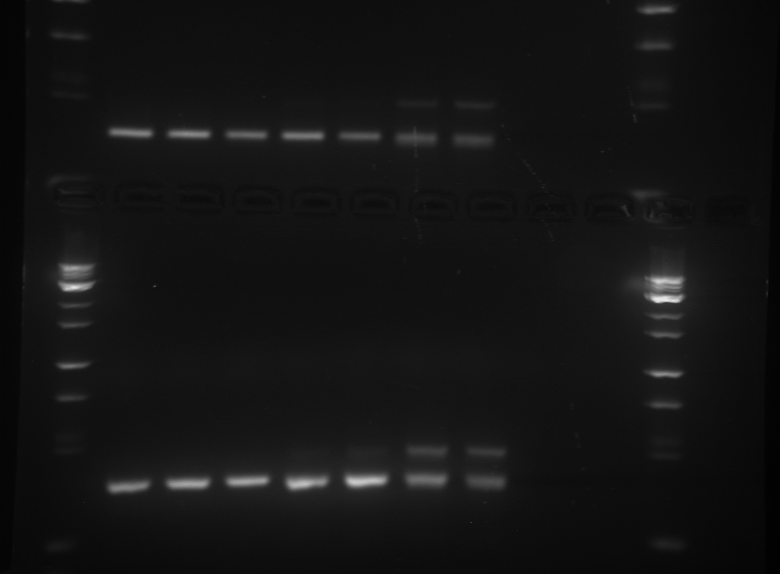

Supplement: Source data 1. — Data file for the raw gels compiled in Source data 2. [file elife-91288-data1.zip › Source Data – File 1 - Raw Gel Images/Figure S3A Integration rate for SF3B1 gene replicate 2.tif]

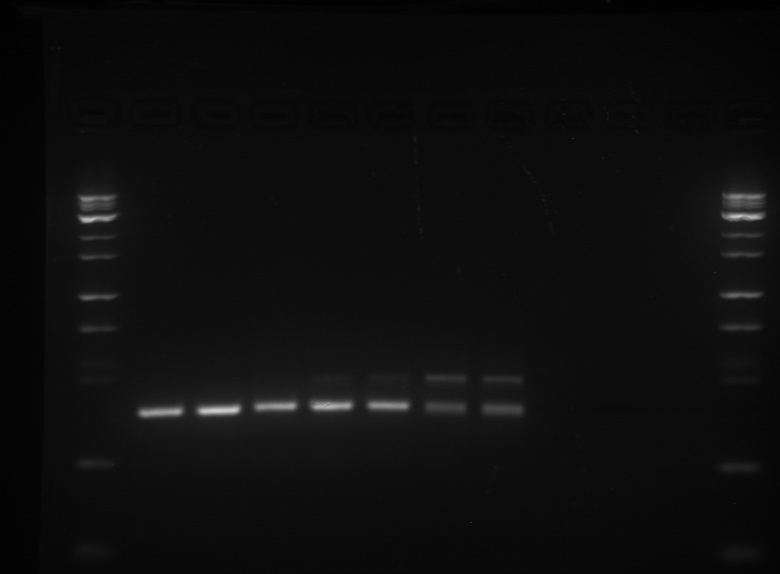

Supplement: Source data 1. — Data file for the raw gels compiled in Source data 2. [file elife-91288-data1.zip › Source Data – File 1 - Raw Gel Images/Figure S3A Integration rate for SF3B1 gene replicate 3.tif]
