# Supplementary material for: Near-perfect precise on-target editing of human hematopoietic stem and progenitor cells
Source: eLife. 2024 Jun 3;12:RP91288. doi: 10.7554/eLife.91288 (PMC11147503; doi:10.7554/eLife.91288)
Supplement: Source data 2. — Compilation of the annotated gels used for quantification of RNP and HDR integration efficiencies. [file elife-91288-data2.pdf]

Source data gels for efficiency assessment:

Figure S1A: SRSF2 RNP efficiency assays:

SRSF2 replicate 1:

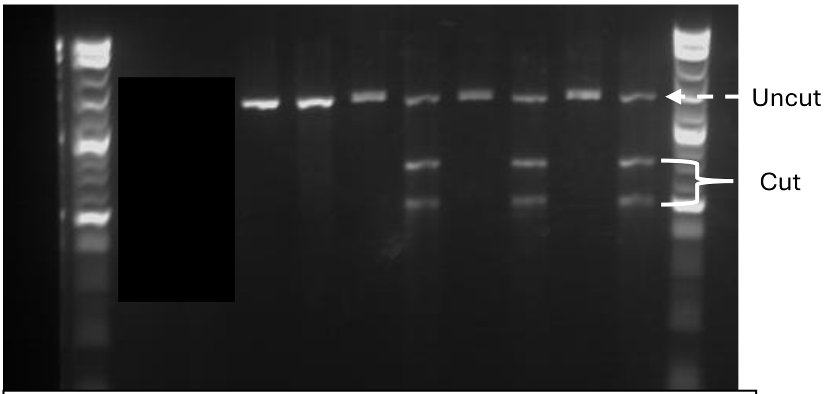

Ladder – Control uncut – Control cut – 30.5pmol uncut – 30.5pmol uncut – 30.5pmol + mol uncut – 30.5pmol + mol cut – 61pmol uncut – 61pmol cut - Ladder

SRSF2 replicate 2:

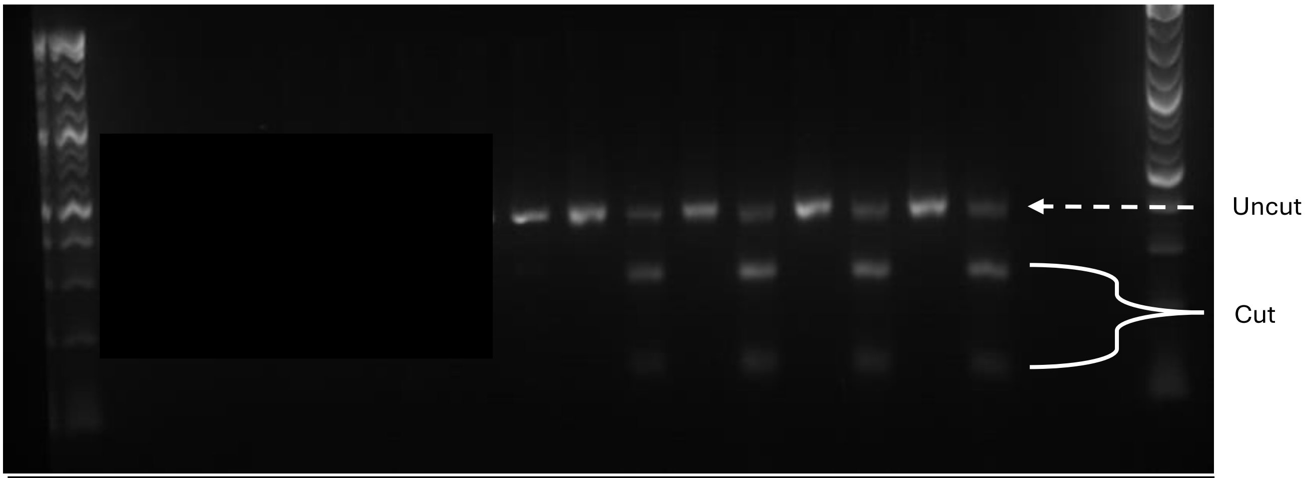

Ladder – Control uncut – Control cut – 30.5pmol uncut – 30.5pmol uncut – 30.5pmol + mol uncut – 30.5pmol + mol cut – 61pmol uncut – 61pmol cut – 61pmol + mol uncut – 61pmol + mol cut - Ladder

SRSF2 replicate 3:

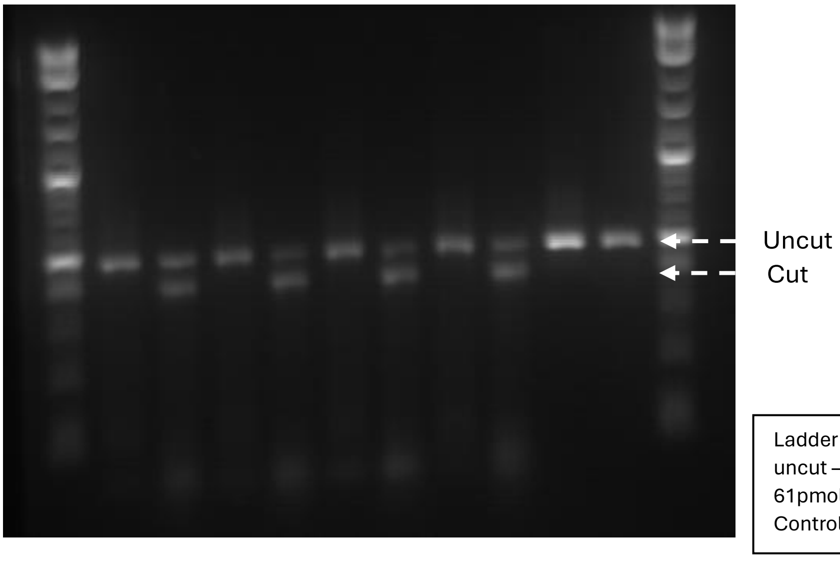

Ladder –30.5pmol uncut – 30.5pmol uncut – 30.5pmol + mol uncut – 30.5pmol + mol cut – 61pmol uncut – 61pmol cut – 61pmol + mol uncut – 61pmol + mol cut - Control uncut – Control cut – Ladder

SRSF2 replicate 4:

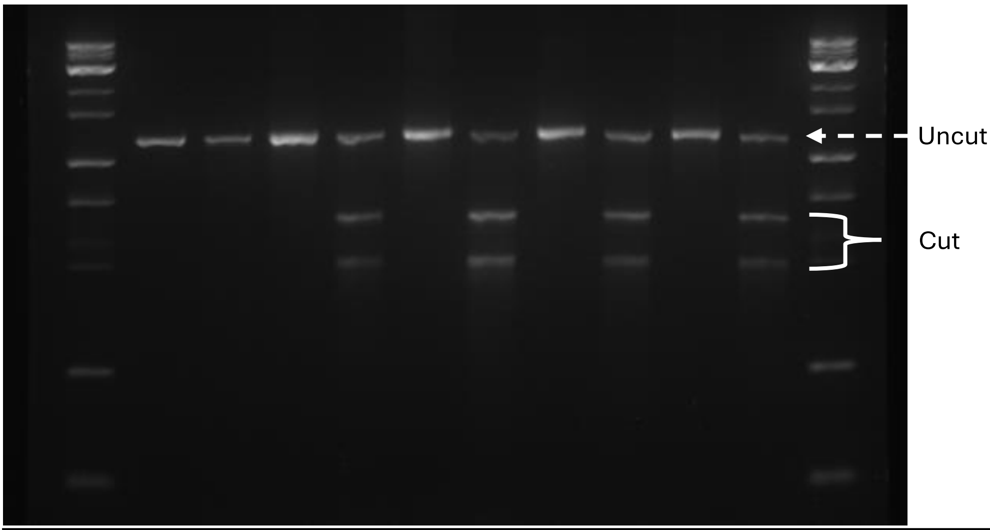

Ladder – Control uncut – Control cut – 30.5pmol uncut – 30.5pmol uncut – 30.5pmol + mol uncut – 30.5pmol + mol cut – 61pmol uncut – 61pmol cut – 61pmol + mol uncut – 61pmol + mol cut - Ladder

SRSF2 replicate 5:

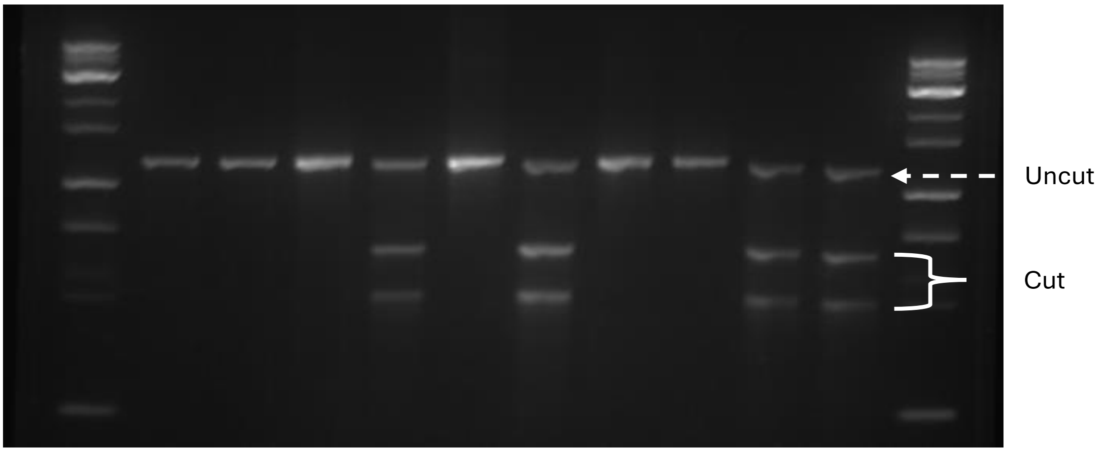

Ladder – Control uncut – Control cut – 30.5pmol uncut – 30.5pmol uncut – 30.5pmol + mol uncut – 30.5pmol + mol cut – 61pmol uncut – 61pmol + mol uncut – 61pmol cut – 61pmol + mol cut - Ladder

Figure 1E: SRSF2 short and long donor assay:

Replicate 1 for short donor:

Ladder – Control unedited – RNP only – short 20pmol – short 20pmol + RNP – Short 50pmol – Short 50pmol + RNP – Short 100pmol – Short 100pmol + RNP – Ladder

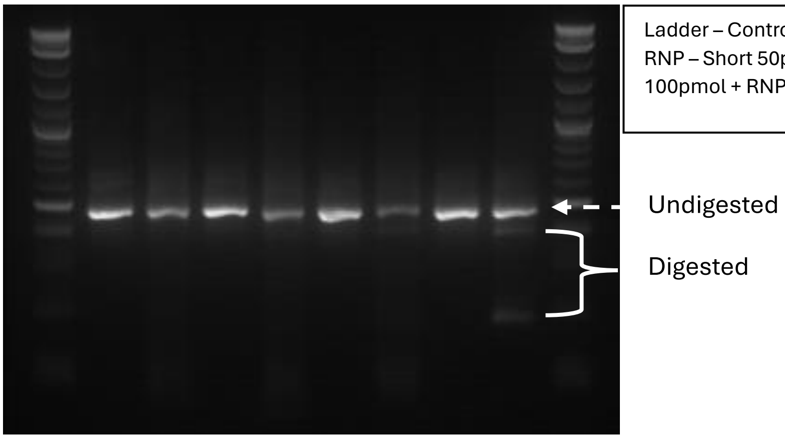

Replicate 2 for short donor and 1 for long donor:

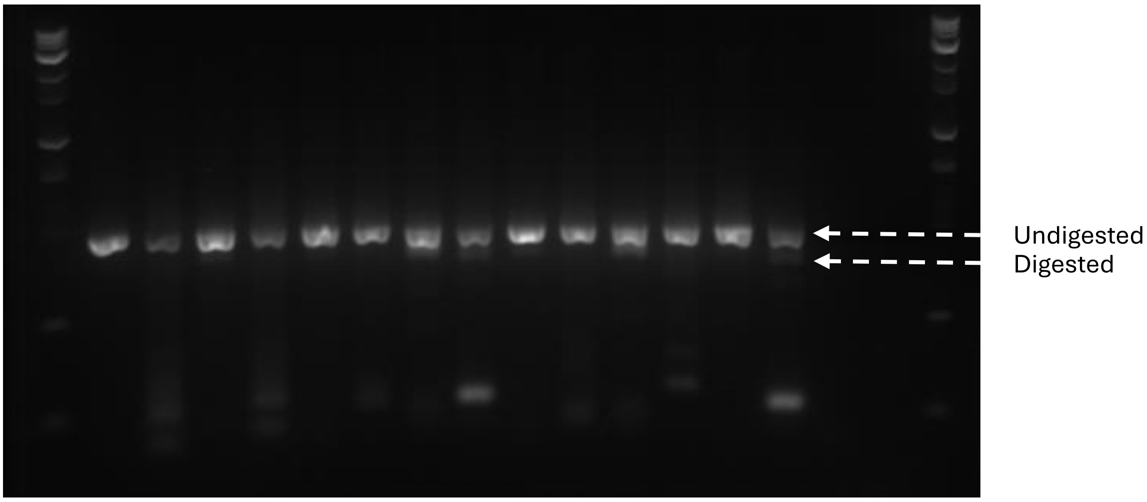

Ladder – Control unedited – RNP only – Long 5pmol – Long 5pmol + RNP – Long 10pmol – Long 10pmol + RNP – Long 20pmol – Long 20pmol + RNP – short 20pmol + RNP - Short 20pmol– Short 50pmol + RNP - Short 50pmol – Short 100pmol – Short 100pmol + RNP – Ladder

Replicate 3 for short donor and 2 for long donor:

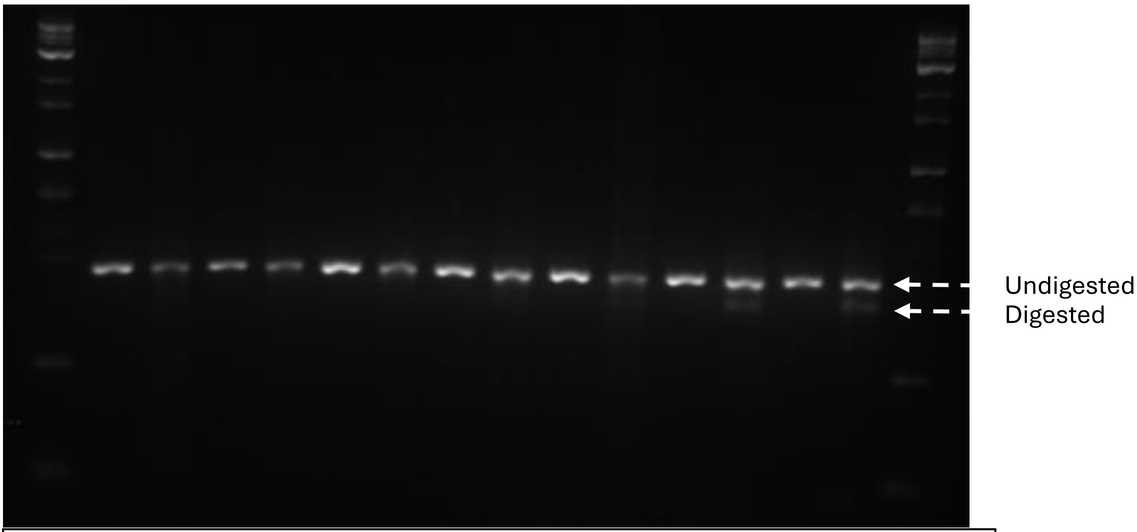

Ladder – Control unedited – RNP only – Long 5pmol – Long 5pmol + RNP – Long 10pmol – Long 10pmol + RNP – Long 20pmol – Long 20pmol + RNP – short 20pmol – short 20pmol + RNP – Short 50pmol – Short 50pmol + RNP – Short 100pmol – Short 100pmol + RNP – Ladder

Replicate 3 for long donor:

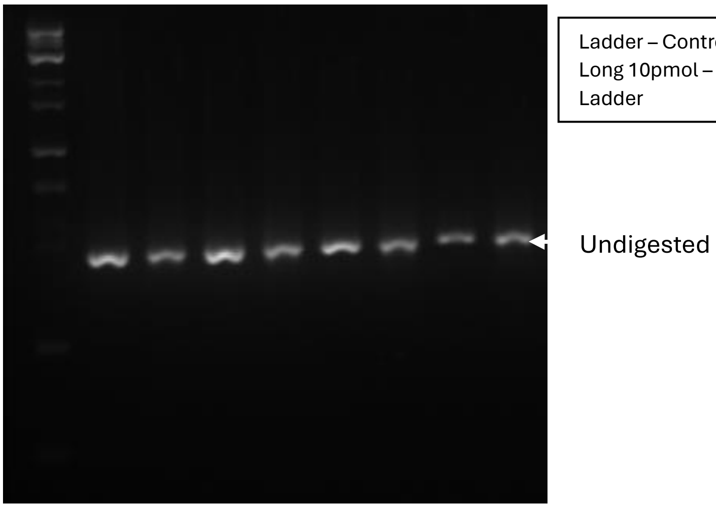

Ladder – Control unedited – RNP only – Long 5pmol – Long 5pmol + RNP – Long 10pmol – Long 10pmol + RNP – Long 20pmol – Long 20pmol + RNP – Ladder

Figure 1C: SRSF2 AAV MOI assay:

AAV MOI replicate 1:

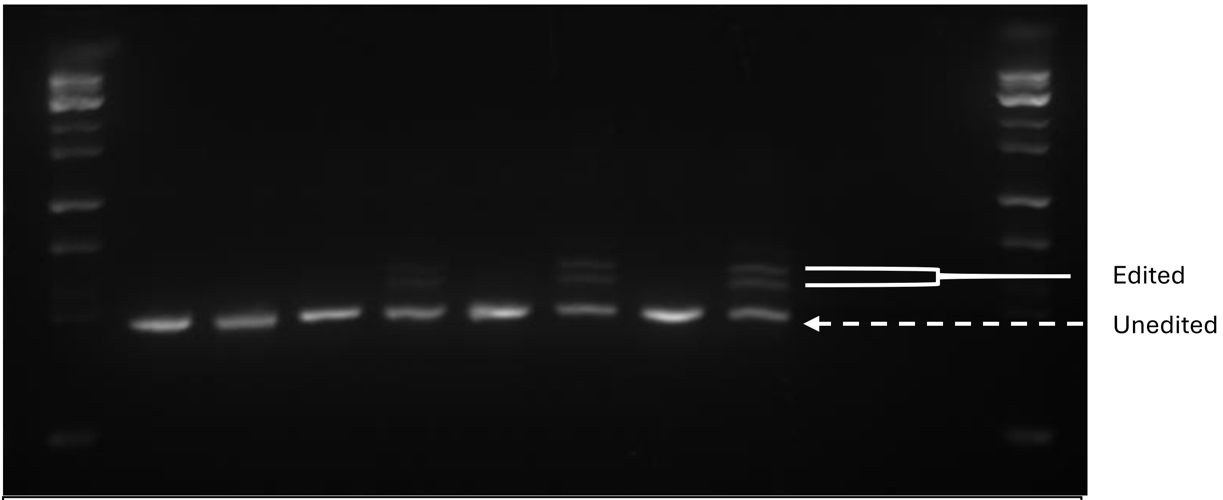

Ladder – Control unedited – RNP only – AAV 200MOI – AAV 200MOI + RNP – AAV 400MOI – AAV 400MOI + RNP – AAV 800MOI – AAV 800MOI + RNP – Control PCR mix 1 – Control PCR mix 2 – Ladder

AAV MOI replicate 2:

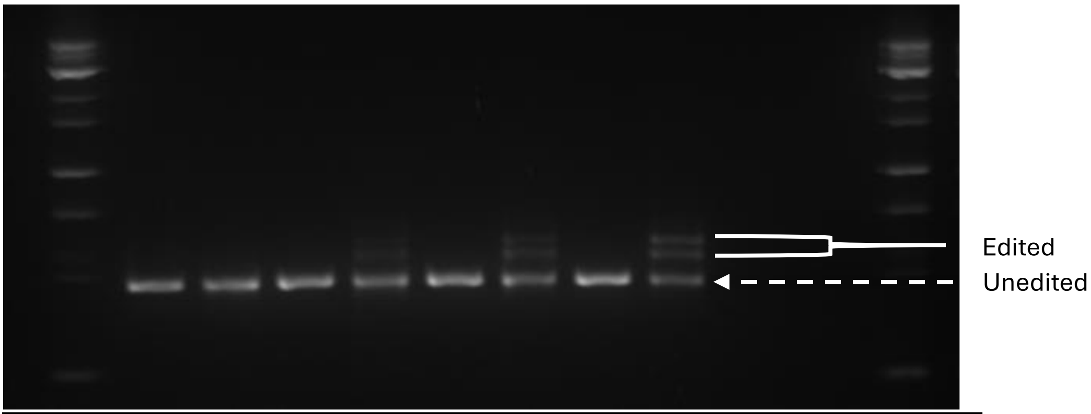

Ladder – Control unedited – RNP only – AAV 200MOI – AAV 200MOI + RNP – AAV 400MOI – AAV 400MOI + RNP – AAV 800MOI – AAV 800MOI + RNP – Control PCR mix 1 – Control PCR mix 2 – Ladder

AAV MOI replicate 3:

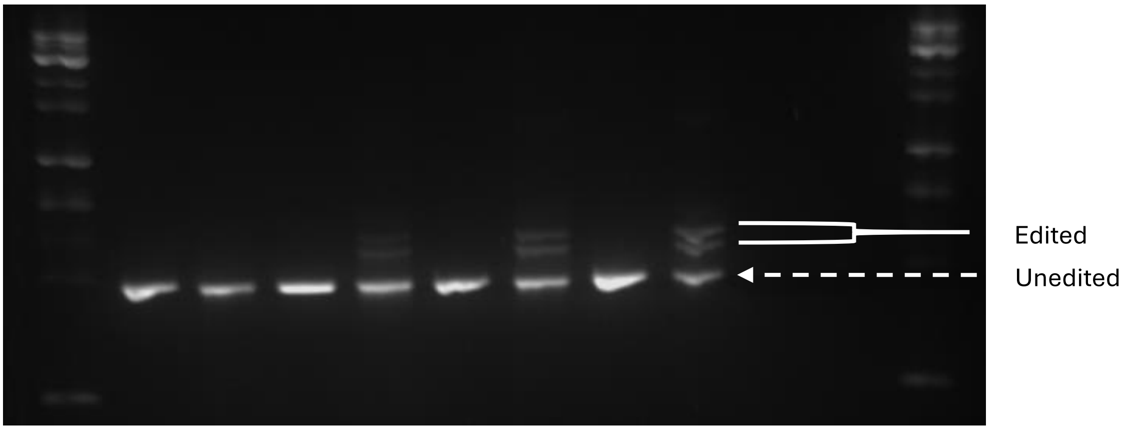

Ladder – Control unedited – RNP only – AAV 200MOI – AAV 200MOI + RNP – AAV 400MOI – AAV 400MOI + RNP – AAV 800MOI – AAV 800MOI + RNP – Control PCR mix 1 – Control PCR mix 2 – Ladder

Figure 2A : AZD7648 vs M3814 comparison assay:

AZD7648 vs M3814 replicate 1:

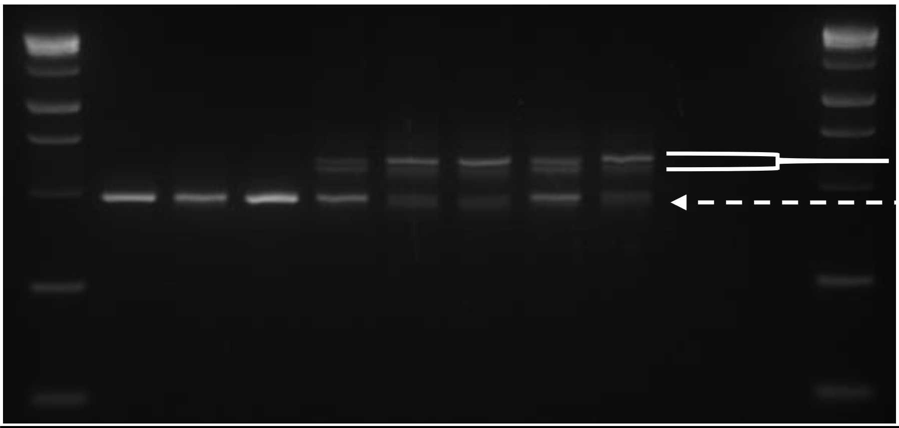

Ladder – Control unedited – RNP only – Donor only – Donor + RNP – Donor + RNP + M3814 0.5μM – Donor + RNP + M3814 5μM – Donor + RNP + AZD7648 0.5μM – Donor + RNP + AZD7648 5μM - Control PCR mix 1 – Control PCR mix 2 – Ladder

AZD7648 vs M3814 replicate 2:

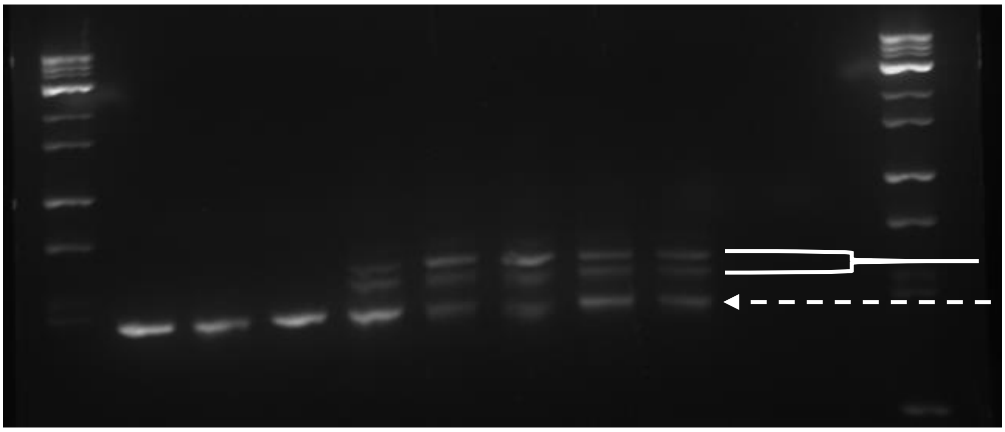

Ladder – Control unedited – RNP only – Donor only – Donor + RNP – Donor + RNP + M3814 0.5μM – Donor + RNP + M3814 5μM – Donor + RNP + AZD7648 0.5μM – Donor + RNP + AZD7648 5μM - Control PCR mix 1 – Control PCR mix 2 – Ladder

AZD7648 vs M3814 replicate 3:

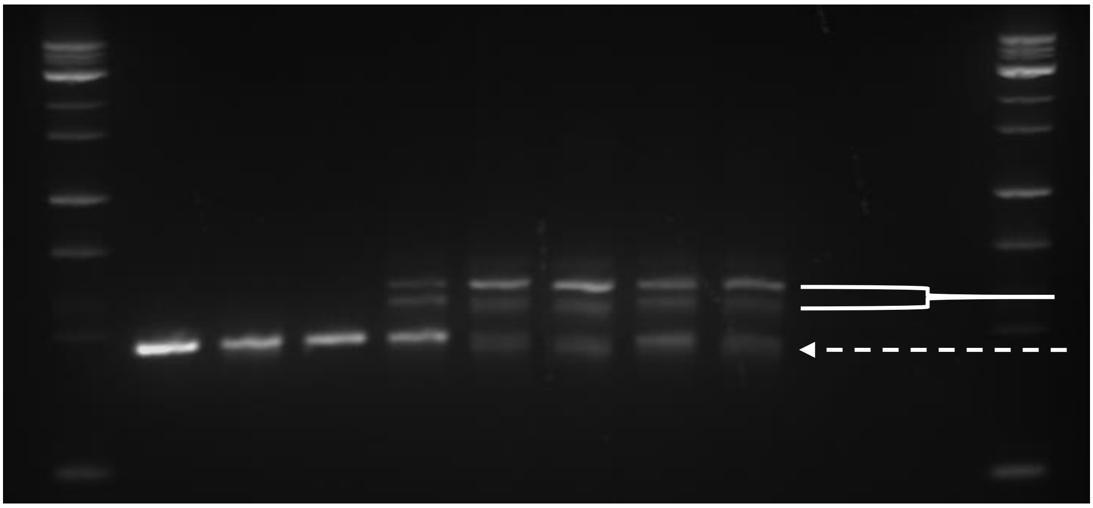

Ladder – Control unedited – RNP only – Donor only – Donor + RNP – Donor + RNP + M3814 0.5μM – Donor + RNP + M3814 5μM – Donor + RNP + AZD7648 0.5μM – Donor + RNP + AZD7648 5μM - Control PCR mix 1 – Control PCR mix 2 – Ladder

Figure 2C: AZD7648 and RS1 additive effect assay:

AZD7648 and RS1 additive effect assay replicate 1:

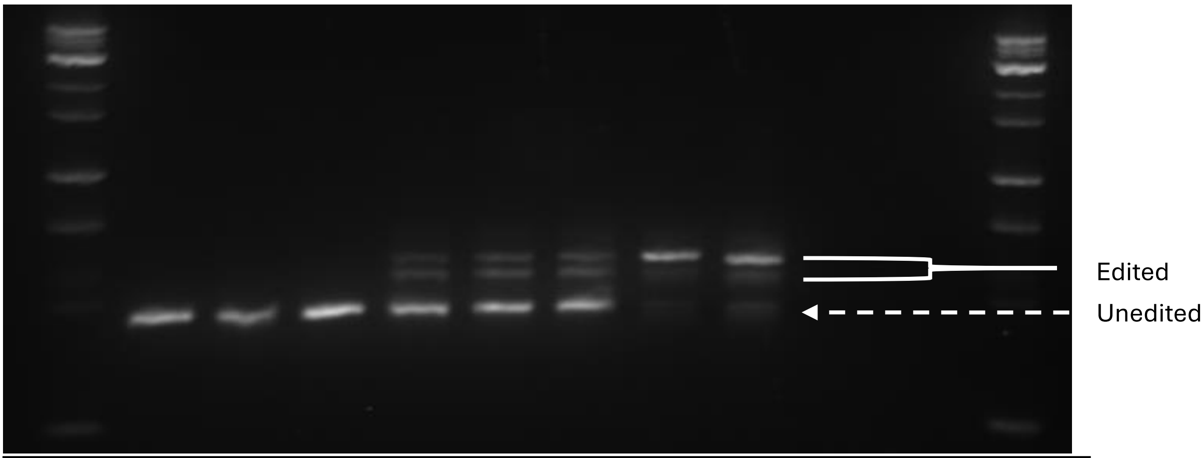

Ladder – Control unedited – RNP only – Donor only – siRNA p53 20fmol only – siRNA p53 20fmol + RS1 15µM – siRNA p53 20fmol + AZD7648 5µm – siRNA p53 20fmol + RS1 15µM + AZD7648 5µM – Control PCR mix 1 – Control PCR mix 2 – Ladder

AZD7648 and RS1 additive effect assay replicate 2:

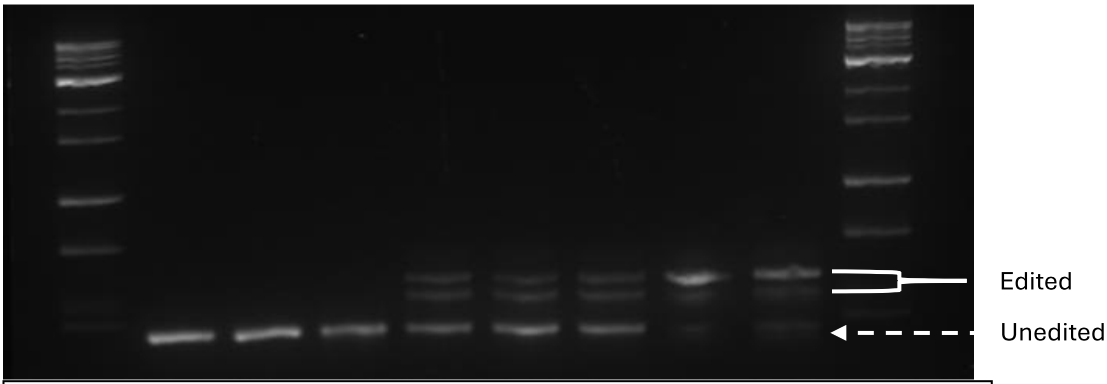

Ladder – Control unedited – RNP only – Donor only – siRNA p53 20fmol only – siRNA p53 20fmol + RS1 15µM – siRNA p53 20fmol + AZD7648 5µm – siRNA p53 20fmol + RS1 15µM + AZD7648 5µM – Ladder

AZD7648 and RS1 additive effect assay replicate 3:

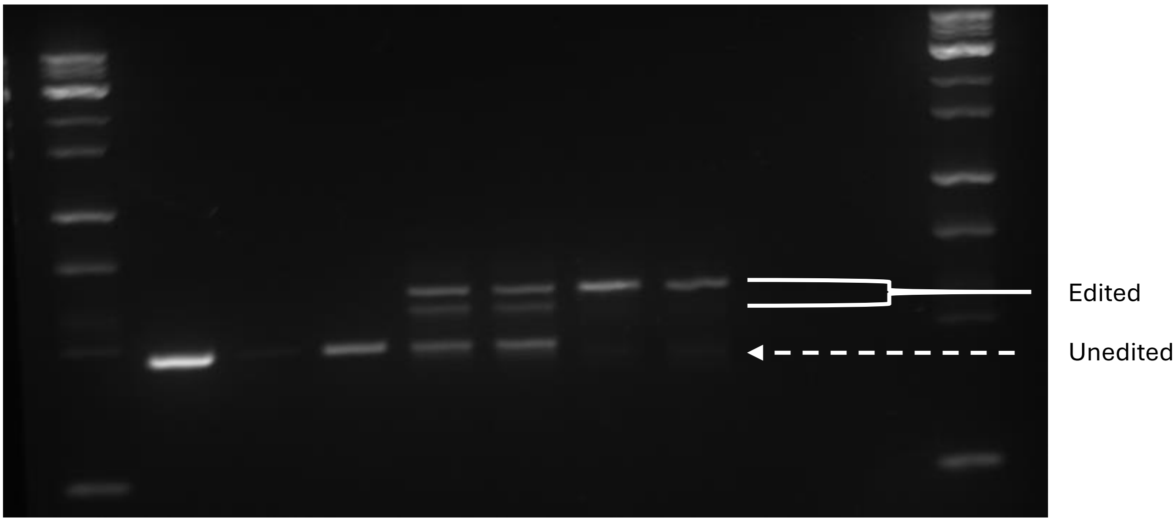

Ladder – Control unedited – RNP only – Donor only – siRNA p53 20fmol only – siRNA p53 20fmol + RS1 15µM – siRNA p53 20fmol + AZD7648 5µm – siRNA p53 20fmol + RS1 15µM + AZD7648 5µM – Control PCR mix 1 – Control PCR mix 2 – Ladder

Figure 4B: Bulk assessment for tuning experiment:

Integration rate in the bulk assessment replicate 1:

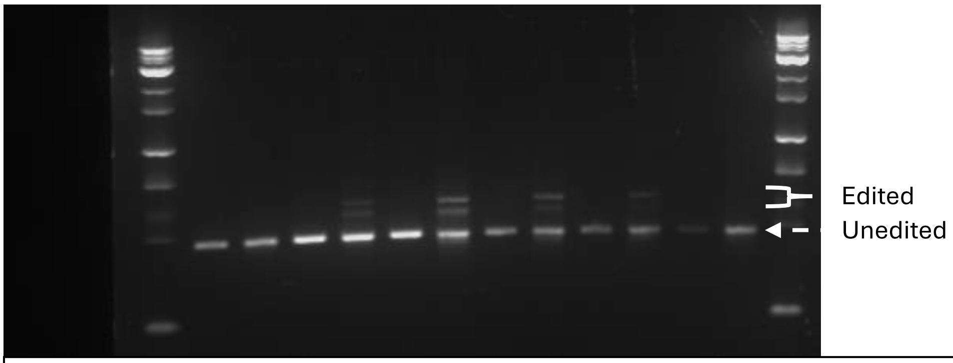

Ladder – Control unedited – RNP only – Donor 100% only – Donor 100% + RNP – Donor 75% only – Donor 75% only + RNP – Donor 50% only – Donor 50% + RNP – Donor 25% only – Donor 25% + RNP – Donor 0% only – Donor 0% + RNP – Ladder

Integration rate in the bulk assessment replicate 2:

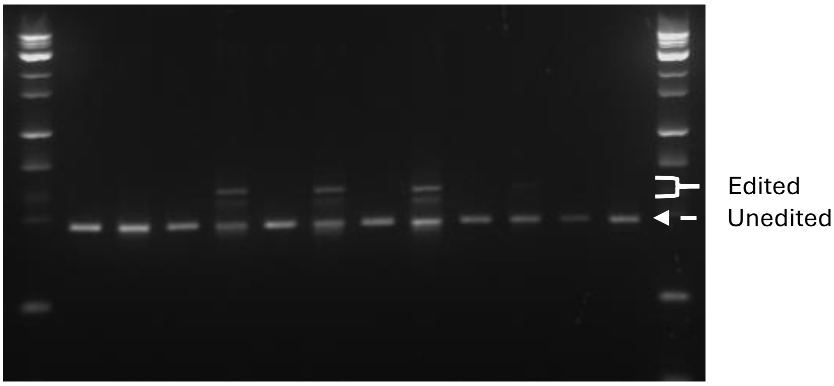

Ladder – Control unedited – RNP only – Donor 100% only – Donor 100% + RNP – Donor 75% only – Donor 75% only + RNP – Donor 50% only – Donor 50% + RNP – Donor 25% only – Donor 25% + RNP – Donor 0% only – Donor 0% + RNP – Ladder

Integration rate in the bulk assessment replicate 3:

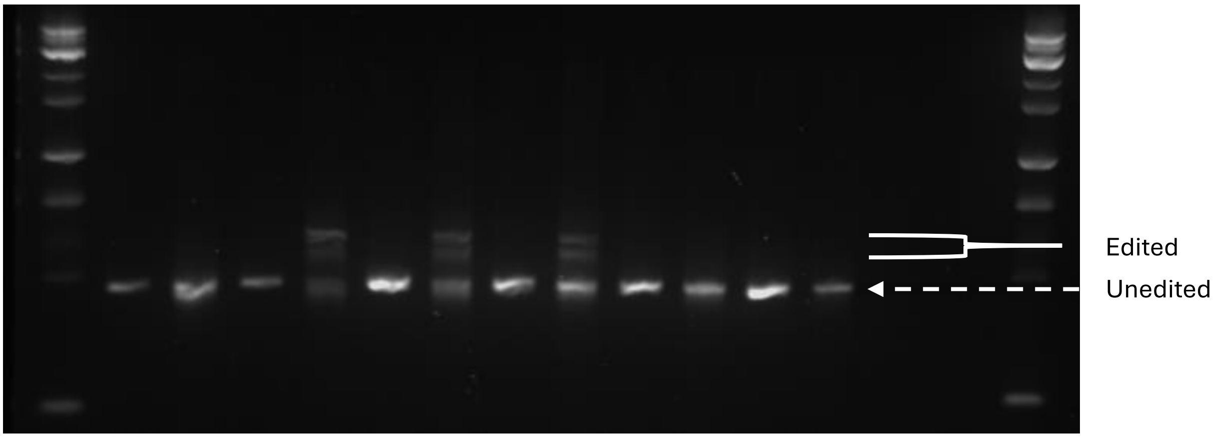

Ladder – Control unedited – RNP only – Donor 100% only – Donor 100% + RNP – Donor 75% only – Donor 75% only + RNP – Donor 50% only – Donor 50% + RNP – Donor 25% only – Donor 25% + RNP – Donor 0% only – Donor 0% + RNP – Control PCR mix 1 – Control PCR mix 2 – Ladder

Integration rate in the bulk assessment replicate 4:

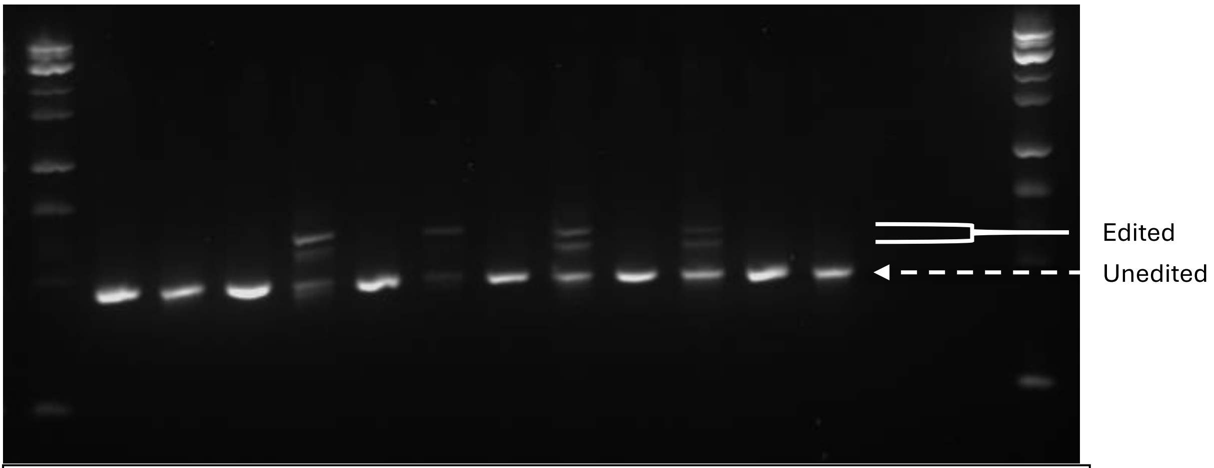

Ladder – Control unedited – RNP only – Donor 100% only – Donor 100% + RNP – Donor 75% only – Donor 75% only + RNP – Donor 50% only – Donor 50% + RNP – Donor 25% only – Donor 25% + RNP – Donor 0% only – Donor 0% + RNP – Control PCR mix 1 – Control PCR mix 2 – Ladder

Figure 3A: Assessment of integration in all 4 sub-populations in CD34+ cells

Integration assessment in all 4 sub-populations replicate 1:

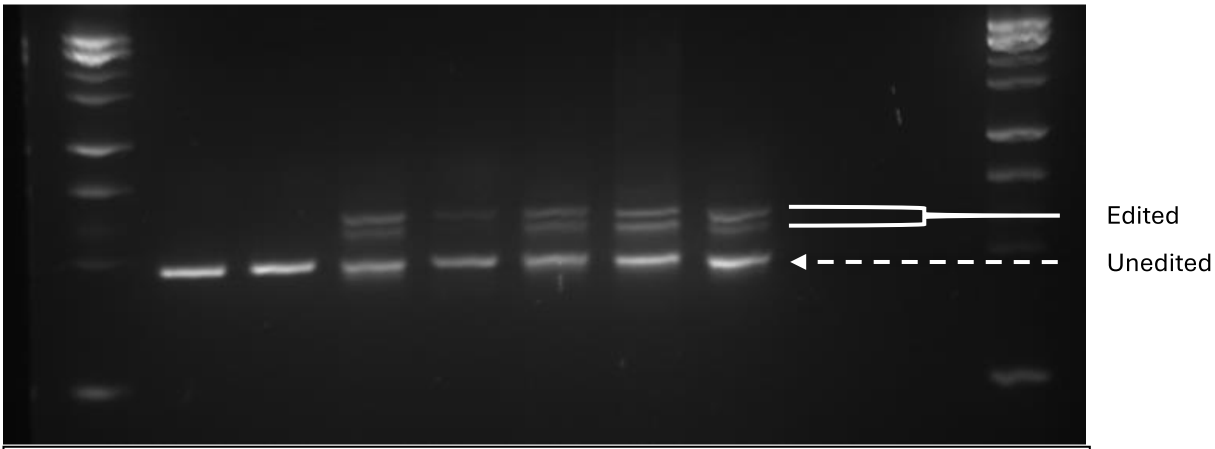

Ladder – Control unedited – Donor only – Bulk – LT-HSCs – lt-HSCs – Multipotent progenitors – Advanced Progenitors – Control PCR mix 1 – Control PCR mix 2 – Ladder

Integration assessment in all 4 sub-populations replicate 2:

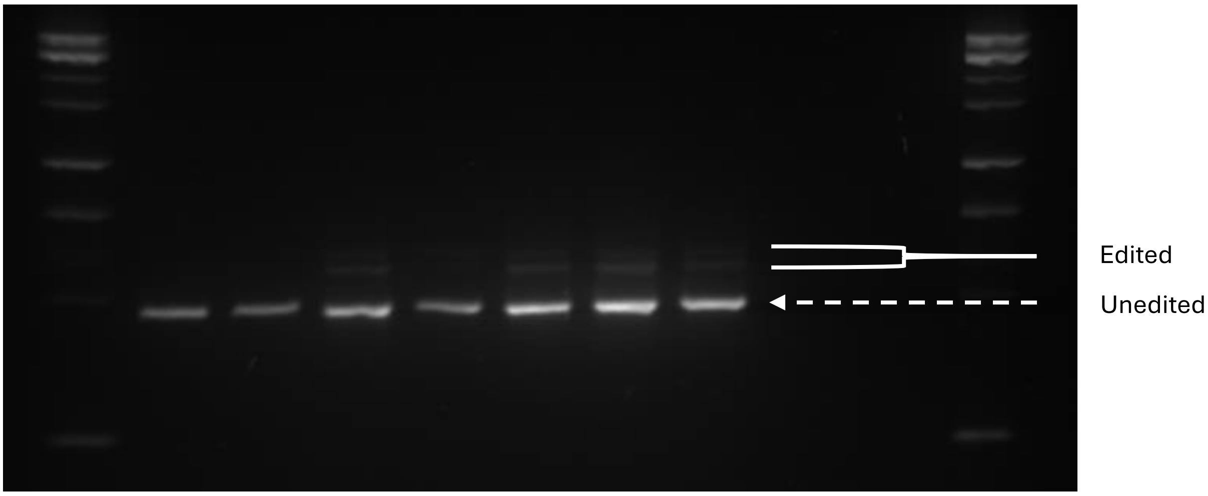

Ladder – Control unedited – Donor only – Bulk – LT-HSCs – lt-HSCs – Multipotent progenitors – Advanced Progenitors – Control PCR mix 1 – Control PCR mix 2 – Ladder

Integration assessment in all 4 sub-populations replicate 3:

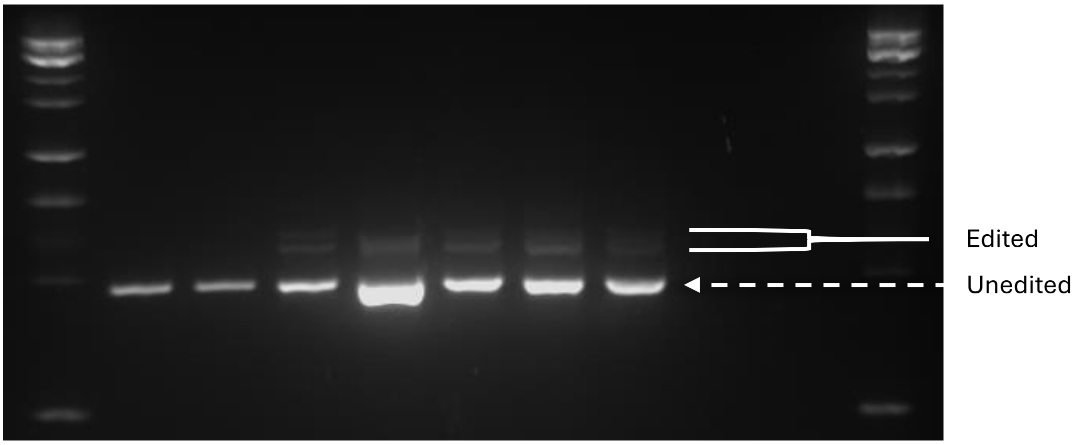

Ladder – Control unedited – Donor only – Bulk – LT-HSCs – lt-HSCs – Multipotent progenitors – Advanced Progenitors – Control PCR mix 1 – Control PCR mix 2 – Ladder

Figure S1C: RNP cutting efficiency for SF3B1 gene:

RNP cutting efficiency for SF3B1 gene replicate 1 and 2:

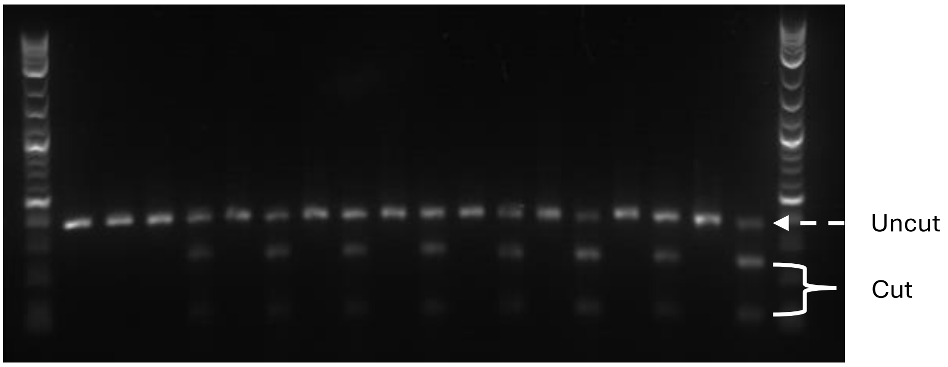

Ladder – Control uncut – Control cut – 30.5pmol uncut (1)– 30.5pmol uncut (1) – 30.5pmol + mol uncut (1) – 30.5pmol + mol cut (1) – 61pmol uncut (1) – 61pmol cut (1) – 61pmol + mol uncut (1) – 61pmol + mol cut (1) – 30.5pmol uncut (2) – 30.5pmol uncut (2) – 30.5pmol + mol uncut (2) – 30.5pmol + mol cut (2) – 61pmol uncut (2) – 61pmol cut (2) – 61pmol + mol uncut (2) – 61pmol + mol cut (2) – Ladder

RNP cutting efficiency for SF3B1 gene replicate 3:

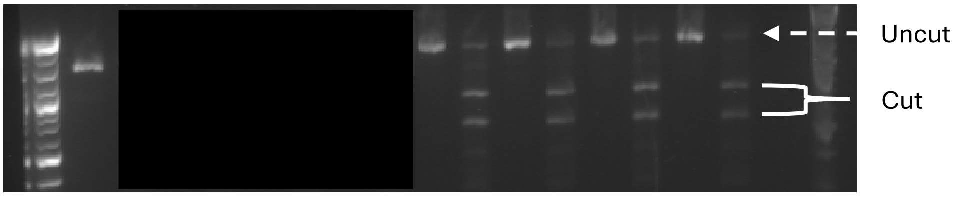

Ladder – Control uncut– 30.5pmol uncut – 30.5pmol uncut – 30.5pmol + mol uncut – 30.5pmol + mol cut – 61pmol uncut – 61pmol cut – 61pmol + mol uncut – 61pmol + mol cut – PCR mix control – Ladder

Figure S3A: Integration rate for SF3B1 gene using AZD7648 and RS1 molecules:

Integration rate for SF3B1 gene replicate 1:

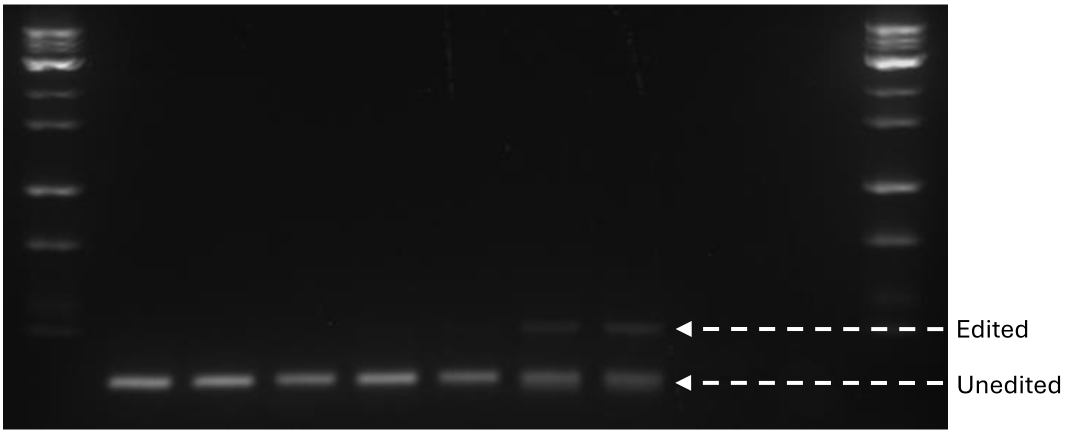

Ladder – Control unedited – RNP only – Donor only – siRNA p53 20fmol only – siRNA p53 20fmol + RS1 15µM – siRNA p53 20fmol + AZD7648 5µm – siRNA p53 20fmol + RS1 15µM + AZD7648 5µM – Control PCR mix 1 – Control PCR mix 2 – Ladder

Integration rate for SF3B1 gene replicate 2:

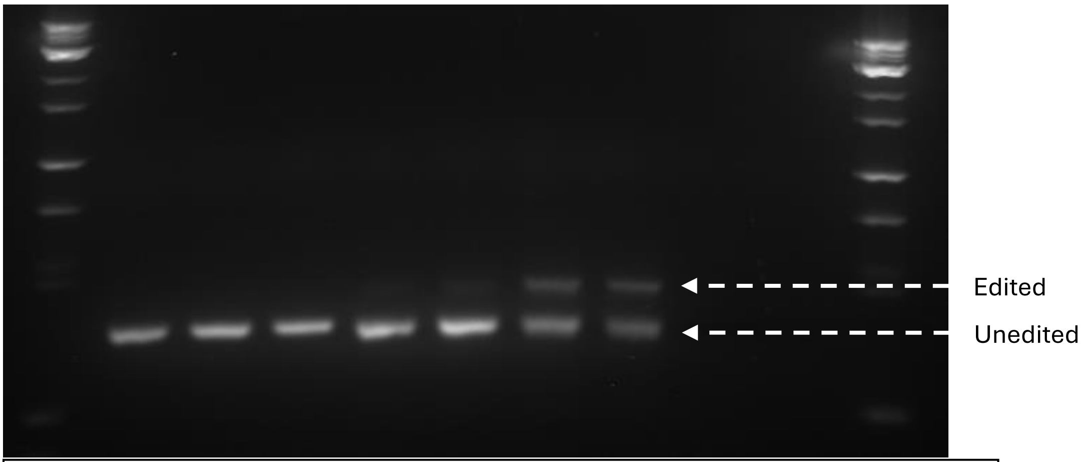

Ladder – Control unedited – RNP only – Donor only – siRNA p53 20fmol only – siRNA p53 20fmol + RS1 15µM – siRNA p53 20fmol + AZD7648 5µm – siRNA p53 20fmol + RS1 15µM + AZD7648 5µM – Control PCR mix 1 – Control PCR mix 2 – Ladder

Integration rate for SF3B1 gene replicate 3:

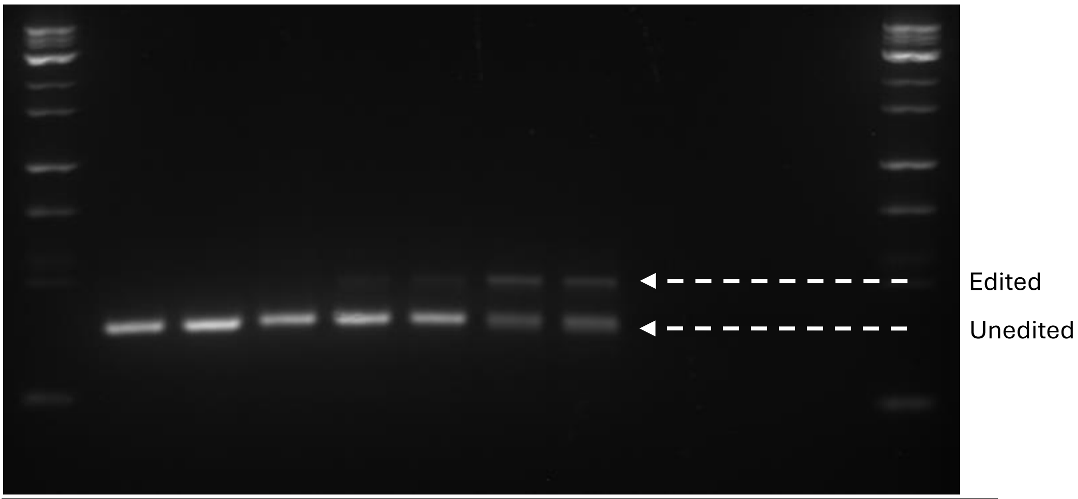

Ladder – Control unedited – RNP only – Donor only – siRNA p53 20fmol only – siRNA p53 20fmol + RS1 15µM – siRNA p53 20fmol + AZD7648 5µm – siRNA p53 20fmol + RS1 15µM + AZD7648 5µM – Control PCR mix 1 – Control PCR mix 2 – Ladder
